# Combined Cytological and Transcriptomic Analysis Reveals a Nitric Oxide Signaling Pathway Involved in Cold-Inhibited *Camellia sinensis* Pollen Tube Growth

**DOI:** 10.3389/fpls.2016.00456

**Published:** 2016-04-14

**Authors:** Weidong Wang, Xianyong Sheng, Zaifa Shu, Dongqin Li, Junting Pan, Xiaoli Ye, Pinpin Chang, Xinghui Li, Yuhua Wang

**Affiliations:** ^1^College of Horticulture, Nanjing Agricultural UniversityNanjing, China; ^2^College of Life Sciences, Capital Normal UniversityBeijing, China

**Keywords:** *Camellia sinensis*, pollen tube growth, nitric oxide, cold stress, signaling pathway

## Abstract

Nitric oxide (NO) as a signaling molecule plays crucial roles in many abiotic stresses in plant development processes, including pollen tube growth. Here, the signaling networks dominated by NO during cold stress that inhibited *Camellia sinensis* pollen tube growth are investigated *in vitro*. Cytological analysis show that cold-induced NO is involved in the inhibition of pollen tube growth along with disruption of the cytoplasmic Ca^2+^ gradient, increase in ROS content, acidification of cytoplasmic pH and abnormalities in organelle ultrastructure and cell wall component distribution in the pollen tube tip. Furthermore, differentially expressed genes (DEGs)-related to signaling pathway, such as NO synthesis, cGMP, Ca^2+^, ROS, pH, actin, cell wall, and MAPK cascade signal pathways, are identified and quantified using transcriptomic analyses and qRT-PCR, which indicate a potential molecular mechanism for the above cytological results. Taken together, these findings suggest that a complex signaling network dominated by NO, including Ca^2+^, ROS, pH, RACs signaling and the crosstalk among them, is stimulated in the *C. sinensis* pollen tube in response to cold stress, which further causes secondary and tertiary alterations, such as ultrastructural abnormalities in organelles and cell wall construction, ultimately resulting in perturbed pollen tube extension.

## Introduction

Low temperature is a major factor that significantly constrains the life cycle of higher plants, including germination, growth, development, flowering, and seed setting (Klemens et al., 2014; Maruyama et al., 2014). Among these processes, reproductive processes, particularly pollen tube growth, are negatively regulated by low temperatures (Hedhly, 2011). According to previous reports, cold stress significantly reduces the pollen tube growth of *Cicer arietinum* (Srinivasan et al., 1999) and *Pyrus bretschneideri* (Gao et al., 2014) and disrupts the morphology of the pollen tube tip zone (Srinivasan et al., 1999). Recently, the actin cytoskeleton, endocytosis and some signaling molecules, such as the calcium ion (Ca^2+^) and reactive oxygen species (ROS), have been implicated in the cold stress-inhibited pollen tube growth *in vitro* (Wu et al., 2012; Gao et al., 2014). However, the underlying basis of the cellular mechanisms of pollen tube growth under cold stress remains largely unknown.

Nitric oxide (NO) is a highly active gaseous signaling molecule that plays crucial roles in many key physiological processes in plants, including seed germination, photo morphogenesis, mitochondrial activity, leaf expansion, root growth, regulation of stomatal movement, fruit maturation, senescence and iron metabolism, etc. (Lanteri et al., 2008; Neill et al., 2008; Sanz et al., 2015). Furthermore, many investigations have revealed that NO plays an important role in the reproductive processes of higher plants, including flower bud differentiation, flowering induction, fertilization and seed setting, particularly pollen tube tip growth (Prado et al., 2008; Domingos et al., 2015). For example, NO participated in self-incompatibility-induced programmed cell death (PCD) in the *Papaver rhoeas* pollen tube through interactions with Ca^2+^, ROS and actin signaling (Wilkins et al., 2011), and there was a suspected potential association between NO and other signaling factors, such as the MAPK cascade and cytoplasmic pH, in this process (Wilkins et al., 2014, 2015). Similarly, NO was found to modulate both the influx of extracellular Ca^2+^ and actin filament organization during cell wall construction to regulate the tip growth of *Pinus bungeana* pollen tubes (Wang et al., 2009). Moreover, NO is also involved in the tolerance of plants to various abiotic stresses (Qiao et al., 2014), such as high salt (Qiao et al., 2009), heat (Xuan et al., 2010), drought (Liao et al., 2012), heavy metals (Saxena and Shekhawat, 2013; Kováčik et al., 2014), and UV-B irradiation stress (Tossi et al., 2009), particularly in cold acclimation and freezing tolerance of plants. For example, Majláth et al. (2012) reported an increased production of NO in *Triticum aestivum* roots after exposure to cold stress, but a decrease of NO content was found in *Capsicum annuum* leaves when they were exposed to low temperatures (Airaki et al., 2012). Recently, NO has been proposed to protect plants from chilling injury by increasing their antioxidant defenses and thereby preventing ROS damage; NO stimulated the activity of S-nitrosylated proteins in *Brassica juncea* under cold stress (Sehrawat et al., 2013a). Additionally, NO participates in cold-triggered root growth inhibition by regulating the content of long-chain bases and the expression of cold-responsive genes (Guillas et al., 2013; Puyaubert and Baudouin, 2014). These data therefore suggest that there is a potential signal regulatory network that depends on NO in a plant's response to cold stress; further investigation is required to clarify the underlying mechanisms of this process.

Above all, NO is thought to act as a core signaling molecule in the cold stress-mediated inhibition of pollen tube growth, and this hypothesis has been supported by physiological and pharmacological findings in our previous research, which showed that NO production from NO synthase (NOS)-like activity decreased cold-responsive pollen germination, inhibited pollen tube growth and reduced proline (Pro) accumulation, partly via the cGMP signaling pathway in *Camellia sinensis* (Wang et al., 2012). However, the role of the NO-dependent complex signaling network, including cGMP, Ca^2+^, ROS, actin, and pH signaling and the cross-talk among them, in the process of cold stress-inhibited pollen tube growth, has not yet been elucidated. In the present study, we also investigated the signal transduction roles of NO during pollen tube elongation in response to cold stress in *C. sinensis*. Specifically, we focused on cold-induced NO that is involved in inhibiting the tip growth of the pollen tube, in addition to several linked cellular features that are essential for the NO signaling pathway under cold tolerance, including the cytoplasmic Ca^2+^ gradient, the ROS concentrations, the acidification of the cytoplasm, the tip ultrastructure, and the composition of the cell wall. Moreover, we also performed the identification of differentially expressed genes in the cold-induced NO signaling pathway in *C. sinensis* pollen tubes, including genes involved in NO synthesis, cGMP, Ca^2+^, ROS, pH, actin, the cell wall, and the MAPK cascade, using transcriptomic analyses, which provided insight into the molecular mechanisms that underlie the above events. These data provide further insights into the regulation of NO signaling in the pollen tube response to cold stress in *C. sinensis*.

## Materials and methods

### Plant material and *in vitro* pollen culture

Mature pollen was collected from “*C. sinensis* (L.) O. Kuntze cv. *Longjingchangye*” tea plants. The pollen was pre-incubated in standard culture medium [containing 30 mM MES, 5% (w/v) sucrose, 0.01% (w/v) H_3_BO_3_, 0.05% (w/v) Ca(NO_3_)_2_·4H_2_O, and 5% (w/v) PEG 4000, pH 6.0] at 25°C in the dark for 30 min *in vitro*. For cold stress treatment, the pre-incubated pollen was transferred and maintained at 4°C in the dark. In addition, NO donor DEA NONOate (25 μM) and NO scavenger 2-(4-carboxyphenyl)-4,4,5,5-tetramethylimidazoline-1-oxyl-3-oxide (cPTIO, 200 μM) were used for the pharmacological treatments. All of the following experiments were performed after 1 h treatments, unless otherwise noted.

### Observation of pollen tube elongation and morphology

To measure the mean length of the pollen tubes, approximately 50 pollen tubes were detected in each of the three replicates at 0.5, 1, and 2 h after different treatments. The morphology of the pollen tubes was examined using a Leica DM2500 biological microscope, and digital images were captured with a Leica DFC290 digital color camera (Leica, Germany).

### Measurement of cytoplasmic Ca^2+^ gradient

The pollen tubes were loaded with the fluorescent Ca^2+^ indicator Fluo-4/AM ester (Life Technologies, Invitrogen, USA) according to Spinelli and Gillespie (2012) with slight modifications. Briefly, after the treatments, the samples were incubated at 25 or 4°C in the dark in culture medium containing 20 μM Fluo-4/AM ester (prepared with DMSO) for 15 min. Then, the pollen tubes were rinsed three times with the corresponding culture medium to wash out excess fluorophore. The fluorescence of at least 20 pollen tubes in each of three replicates was detected using a 488-nm argon laser attached to a Laser Scanning Confocal Microscope (LSCM, Zeiss LSM 780, Germany) with the same parameter settings, and emission signals were collected at 515 nm. Image analysis was performed with pseudo color technology (Rainbow2) in ZEN 2013 software.

### Measurement of cytoplasmic ROS

The presence of ROS in the pollen tubes was assayed and visualized with CM-H_2_DCF-DA (Invitrogen, USA) as described by Wilkins et al. (2011) with slight modifications. In brief, the samples were incubated in 5 μM CM-H_2_DCF-DA for 15 min in the dark; then, the excess fluorescent indicator was washed out. The specimens were mounted and photographed with a Zeiss LSM 780 LSCM (excitation at 488 nm and emission at 515 nm). To allow comparisons between images, identical parameter settings were used throughout each experiment. The quantification of relative fluorescence units of at least 20 pollen tubes in each of three replicates was performed using the ImageJ software package, and the mean relative fluorescence intensities were calculated.

### Measurement of cytoplasmic pH

Intracellular [pH]_cyt_ was determined in the living pollen tubes with BCECF AM (Invitrogen, USA) as described by Wilkins et al. (2015) with slight modifications. The pollen tubes were loaded with 2.5 μM BCECF AM for 15 min followed by washing with the corresponding culture medium. The pollen tubes were only imaged within 5 to 10 min after the addition of BCECF AM because this time frame allowed for accurate reporting of [pH]_cyt_. The images of at least 20 pollen tubes in each of three replicates were captured using a Zeiss LSM 780 LSCM with sequential excitation at 488 nm and emission at 510–550 nm, and the image analysis was performed with pseudo color technology (Rainbow2) in ZEN 2013 software.

### Ultrastructure observation with a transmission electron microscope (TEM)

A TEM analysis was performed according to Wang et al. (2014) and Sheng et al. (2006) with slight modifications. The pollen tubes were collected after treatment for 1 h and then fixed in 2.5% glutaraldehyde in 100 mM PBS (pH 7.2) at 4°C for 4 h. Then, they were washed with 100 mM PBS and post-fixed with 2% OsO_4_ for 2 h, washed again, dehydrated in an ethanol series (50, 70, 90, and 100%) and finally embedded in Spurr's epoxy resin. Sections were cut with an LKB-V ultramicrotome, stained with 2% uranyl acetate (w/v) in 70% methanol (v/v), and 0.5% lead citrate and observed using a TEM (H-7650, Hitachi High-technologies Corporation, Japan) at 80 kV.

### Fluorescent immunolabeling of pectins and AGPs in the pollen tube cell wall

The immunolabeling of pectins and AGPs in pollen tube cell walls was performed with LM19, LM20 and LM2 antibodies (PlantProbes, Leeds, UK) according to Chen et al. (2009) with slight modifications. Pollen tubes that had been treated for 1 h were collected and fixed in 4% paraformaldehyde in 100 mM phosphate buffer solution (PBS, pH 7.2) for 30 min at room temperature and rinsed three times for 5 min each with PBS. Subsequently, the specimens were incubated for 2.5 h at room temperature with primary antibodies against acidic pectin (LM19), esterified pectin (LM20) and AGPs (LM2) at a dilution of 1:5. After incubation, the pollen tubes were washed with PBS three times for 10 min each, incubated with a secondary antibody, fluorescein isothiocyanate (FITC)-labeled sheep anti-rat IgG (KPL, Inc. USA), diluted 1:50 with PBS for at least 2 h at room temperature and then washed with PBS three times. The samples were mounted, and then at least 20 pollen tubes in each of three replicates were observed and photographed with a Zeiss LSM 780 LSCM (excitation at 488 m and emission at 522 nm).

### Total RNA extraction and transcriptomic analysis

Pollen was pre-incubated in standard culture medium at 25°C in the dark for 30 min, followed by the various treatments, including the control (25°C, CK), cold stress (4°C, LT) and NO donor (25 μM DEA NONOate, NO), for 1 h in the dark. After incubation, the pollen tubes were collected with a nylon mesh screen (200 meshes) to exclude ungerminated pollen grains; then, they were immediately subjected to grinding in liquid nitrogen. Total RNA from the pollen tubes of three independent experiments (CK1, LT1 and NO1; CK2, LT2 and NO2; and CK3, LT3 and NO3) was extracted using RNAiso Plus (TaKaRa, Japan), and the quality of the total RNA was verified using a 2100 Bioanalyzer RNA Nano chip device (Agilent, Santa Clara, CA, USA) and a NanoDrop ND-1000 spectrophotometer (NanoDrop, Wilmington, DE). The cDNA libraries were constructed and sequenced using an Illumina HiSeq™2000 located at the Beijing Genomics Institute (Shenzhen, China; http://www.genomics.cn/index). To compare the differences in gene expression, a stringent cutoff (a probability > 0.7 and a |log_2_Ratio| ≥ 1.0), was used to identify differentially expressed genes (DEGs).

### Quantitative real-time PCR (qRT-PCR) analysis

Total RNA was isolated from pollen tubes that were subjected to the various treatments described above using RNAiso Plus (TaKaRa, Japan) and treated with DNase I to remove any genomic DNA contamination. The quality of the total RNA was measured with the NanoDrop ND-1000 spectrophotometer (NanoDrop, Wilmington, DE), and the first cDNA strand was synthesized using the PrimeScript^TM^ RT Reagent Kit with gDNA Eraser (TaKaRa, Dalian, China). The qRT-PCR was performed using SYBR® Premix Ex TaqTM II (TaKaRa, Dalian, China) on an Eppendorf Real-Time PCR System (Mastercycler®ep realplex, Germany) according to the manufacturer's protocol. The amplification regimen was set up as described by Ren et al. (2014), and three biological replicates were performed for each sample. Relative expression levels were calculated by including the *C. sinensis* 18 sRNA gene as the reference based on the 2^−ΔΔCT^ method (Livak and Schmittgen, 2001). Primers used for the qRT-PCR are listed in Table S1.

### Statistical analysis

All data are expressed as the means ± standard deviations (SD) obtained from at least three independent replicates. Statistical significance was calculated by one-way ANOVA using Duncan's test in the SPSS 20 software, and the significant differences among various treatment groups are represented by different letters (*P* < 0.05).

## Results

### Pollen tube growth and morphological observations

**Figure 1:**
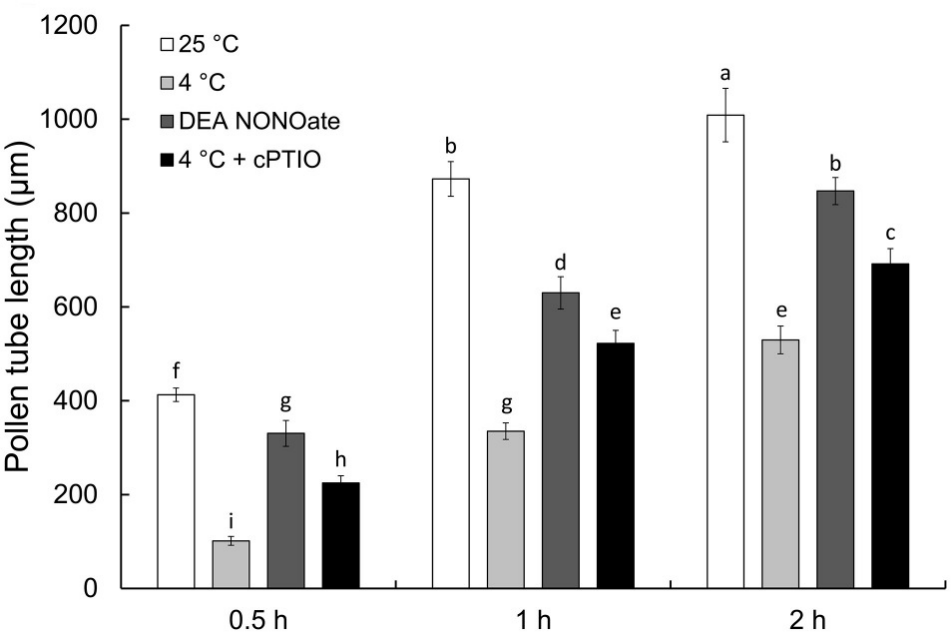
**Effects of cold stress or DEA NONOate on the growth of *C. sinensis* pollen tube**. The growth of pollen tubes was significantly delayed after treatment with cold stress (4°C) or 25 μM DEA NONOate, and the effects of cold stress were reduced by 200 μM cPTIO. The values are the means of three replicates ± SD (*n* ≥ 50). Different letters on bars denote significant differences at *P* < 0.05 according to Duncan's test.

As shown in Figure 1, cold stress and NO donor DEA NONOate significantly delayed pollen tube growth after the 0.5, 1, and 2 h treatments. In addition, the NO scavenger cPTIO was used to confirm the role of NO in the cold stress-induced inhibition of pollen tube growth. Interestingly, the inhibitory effects of cold stress on pollen tube growth were markedly relieved by the simultaneous presence of cPTIO. Furthermore, under control conditions, the pollen tube showed a uniform diameter and a clear zone at the tip (Figure S1A), whereas the pollen tube exhibited obvious abnormalities such as a swollen tip and a loss of the clear zone at the tube tip after cold stress (Figure S1B) or DEA NONOate treatment for 1 h (Figure S1C). Moreover, the effects of cold stress on the morphology of the pollen tube tip were reduced by cPTIO (Figure S1D).

### Effects of cold stress and no on the cytoplasmic Ca^2+^ gradient in the pollen tube tip

**Figure 2:**
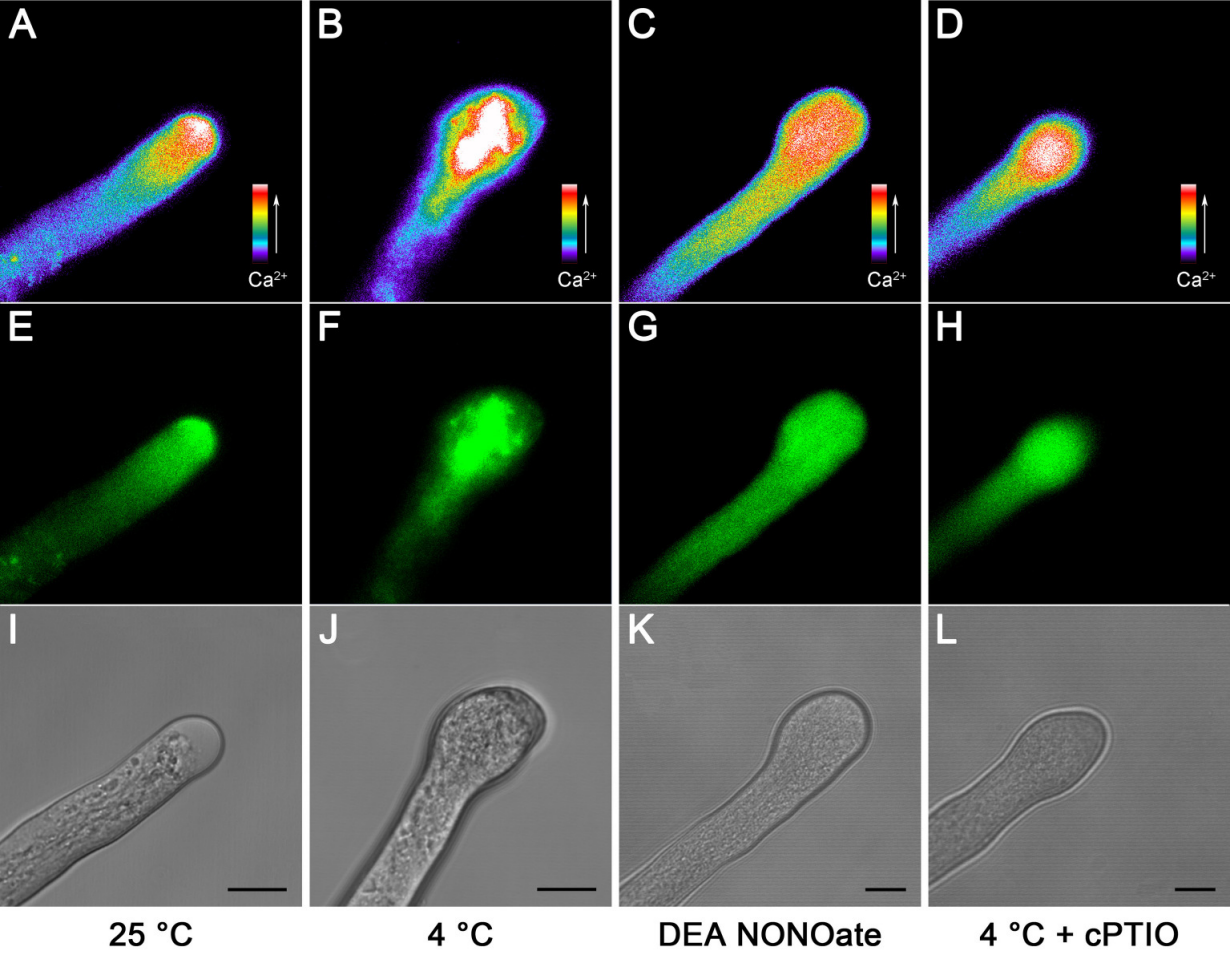
**Changes in the tip-focused Ca^2+^ gradient in *C. sinensis* pollen tubes after treatment with cold stress or DEA NONOate**. Pollen tubes were loaded with the fluorescent Ca^2+^ indicator Fluo-4/AM ester, and the fluorescence was detected using LSCM (488 nm excitation and 515 emission). The control pollen tubes exhibited the typical tip-focused cytoplasmic Ca^2+^ concentration gradient **(A)**. The corresponding fluorescent image **(E)** and bright field image **(I)** are shown below. The pollen tubes that were treated with cold stress **(B)** or 25 μM DEA NONOate **(C)** showed the disrupted cytoplasmic Ca^2+^ gradient. The corresponding fluorescent images **(F,G)** and bright field images **(J,K)** are shown below. The effects of cold stress on the cytoplasmic Ca^2+^ gradient were weakened by 200 μM cPTIO **(D)**. The corresponding fluorescent image **(H)** and bright field image **(L)** are shown. At least 20 pollen tubes were observed and photographed in each of three replicates, and one representative image per treatment was displayed. Bar = 10 μm.

Because Ca^2+^ plays a central role in the tip growth of pollen tubes, the tip-focused Ca^2+^ gradient is also necessary for structural organization of the cytoskeleton in angiosperm pollen tubes (Sheng et al., 2006). Pollen tubes were loaded with Fluo-4/AM to test the possible effects of cold stress and NO treatments on the Ca^2+^ distribution. The results showed that pollen tubes grown under normal conditions exhibited the typical tip-to-base cytoplasmic Ca^2+^ concentration gradient (Figures 2A,E,I), whereas this tip-focused Ca^2+^ gradient was disrupted after cold stress treatment, and stronger fluorescence erratically filled the entire tip of the pollen tubes (Figures 2B,F,J). Similarly, treatment with 25 μM DEA NONOate also led to the disruption of the cytoplasmic Ca^2+^ gradient (Figures 2C,G,K). In contrast, the disruption degree of cytoplasmic Ca^2+^ gradient in the pollen tubes treated with 200 μM cPTIO upon cold stress was relieved (Figures 2D,H,L) compared with that in the pollen tubes treated with cold stress alone (Figures 2B,F,J).

### Cytoplasmic ROS is increased in the pollen tube by cold stress and no

**Figure 3:**
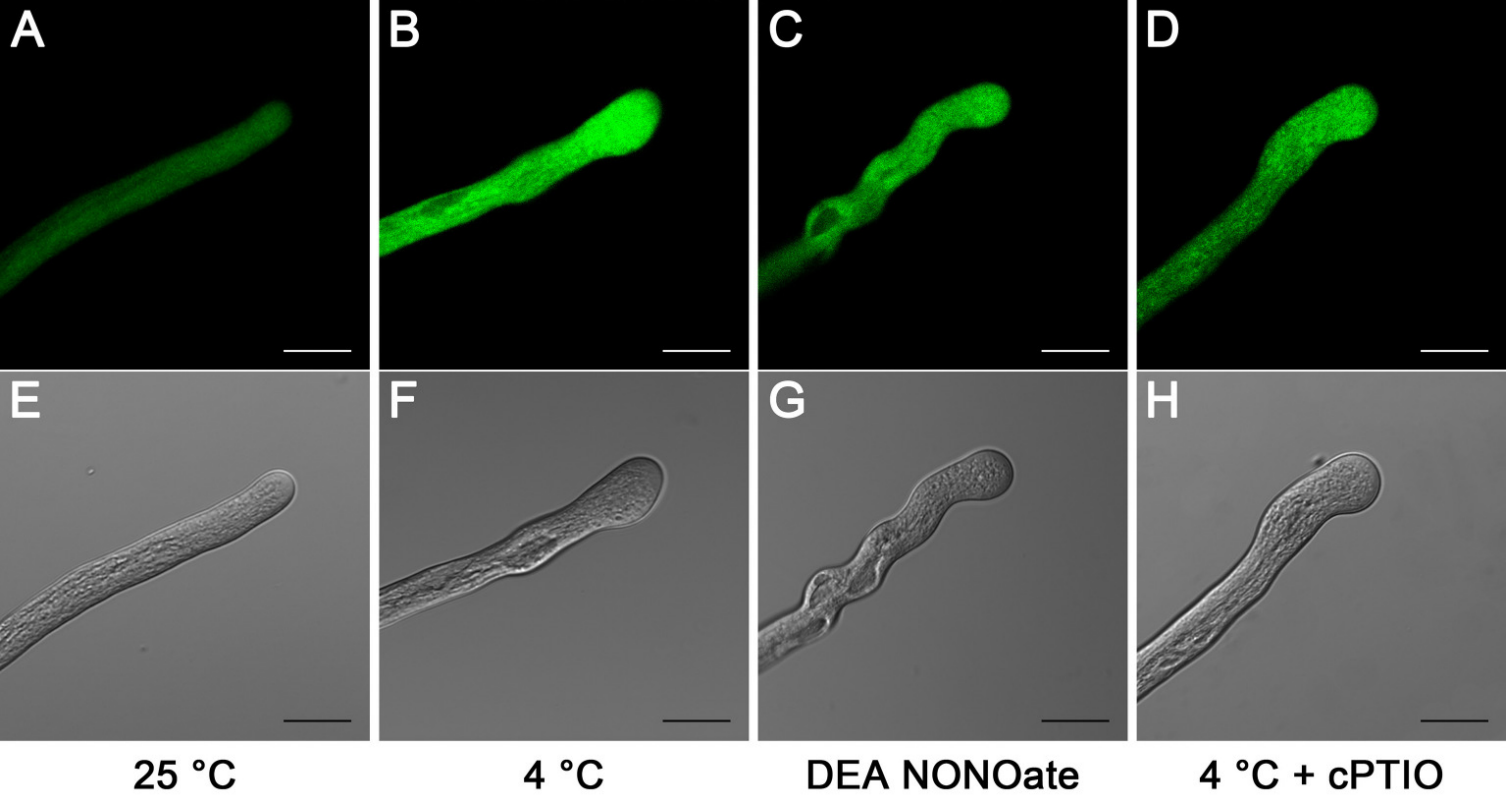
**Effects of cold stress or DEA NONOate on cytoplasmic ROS in *C. sinensis* pollen tubes**. In the control pollen tubes, the ROS fluorescence signal was symmetrical and weak and was distributed throughout almost the entire pollen tube **(A)**. After treatment with cold stress **(B)** or 25 μM DEA NONOate **(C)** for 1 h, the ROS fluorescence signal significantly increased, particularly in the tip region. The increase in the ROS fluorescence signal stimulated by cold stress was largely reduced after treatment with 200 μM cPTIO **(D)**. The corresponding bright field images are shown below **(E–H)**. At least 20 pollen tubes were observed and photographed in each of three replicates, and one representative image per treatment was displayed. Bar = 20 μm.

To examine the relative levels of endogenous ROS, pollen tubes were labeled with CM-H_2_DCF-DA, and ROS were monitored using a LSCM. In the control, the ROS fluorescence signal was distributed evenly throughout the entire pollen tube (Figures 3A,E). In comparison, the ROS fluorescence signal was significantly increased after 1 h of cold stress treatment, particularly in the tip region (Figures 3B,F). Similarly, a higher intensity ROS fluorescence signal was detected in the pollen tubes that were treated with 25 μM DEA NONOate (Figures 3C,G) than that in the controls (Figures 3A,E). Furthermore, the ROS fluorescence signal was weaker in the pollen tubes that were treated with 200 μM cPTIO under cold stress (Figures 3D,H) than in those treated with cold stress alone (Figures 3B,F), and the fluorescence signal (Figures 3D,H) was stronger than that in the controls (Figures 3A,E). Similarly, the quantification analysis showed that the average fluorescence intensity of the ROS was significantly increased by cold stress or DEA NONOate by 3.07-fold and 1.86-fold compared with the control, respectively, and the increase in the average fluorescence intensity induced by cold stress was decreased 2.18-fold with 200 μM cPTIO (Figure S2). These data suggest that increases in NO can stimulate increases in ROS under cold stress in pollen tubes of *C. sinensis*.

### Dramatic acidification of the pollen tube tip is induced by cold stress and no

**Figure 4:**
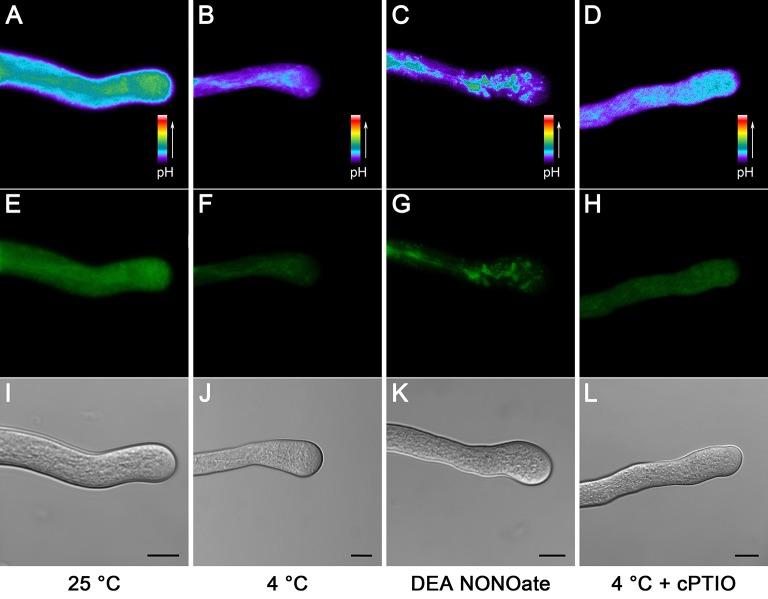
**Cold stress or DEA NONOate induces cytoplasmic pH ([pH]_*cyt*_) acidification in *C. sinensis* pollen tubes**. Pollen tubes [pH]_cyt_ were labeled with the pH indicator BCECF AM prior to imaging using LSCM. Compared with the control pollen tubes **(A)**, the levels of the fluorescence signal were significantly decreased after 1 h of cold stress **(B)** or 25 μM DEA NONOate **(C)** treatments, and the degree of pollen tube cytoplasmic acidification mediated by cold stress was significantly reduced after treatment with 200 μM cPTIO **(D)**. The corresponding fluorescent images **(E–H)** and bright field images **(I–L)** are shown below, respectively. At least 20 pollen tubes were observed and photographed in each of three replicates, and one representative image per treatment was displayed. Bar = 10 μm.

Pollen tube [pH]_cyt_ (cytoplasmic pH) has been demonstrated to play a vital role in pollen tube growth (Michard et al., 2008; Wilkins et al., 2015). We therefore investigated cold stress-induced [pH]_cyt_ changes using the ratiometric pH indicator 2,7-bis-(2-carboxyethyl)-5-(and-6)-carboxy fluorescein (BCECF) acetoxymethyl ester (AM). The results showed that the [pH]_cyt_ decreased after 1 h of cold stress treatment (Figures 4B,F,J) compared to that in the control pollen tubes (Figures 4A,E,I), implying that cold stress induced the acidification in the pollen tube tip zone. Similarly, the levels of the fluorescence signal were also decreased after treatment with 25 μM DEA NONOate (Figures 4C,G,K). To examine whether NO is involved in cold-induced cytoplasmic acidification, 200 μM cPTIO was used to treat pollen tubes under cold stress treatment, and the [pH]_cyt_ was examined. The results showed that the degree of pollen tube cytoplasmic acidification was significantly reduced, although this reduction did not completely reverse the effects of the cold stress on the pollen tube [pH]_cyt_ (Figures 4D,H,L). These data show the functional importance of NO in the process of cold stress-induced pollen tube cytoplasmic acidification and provide insight into the mechanisms of NO involved in cold-inhibited pollen tube growth.

### Cold stress and no disrupt the distribution of organelles and induce cell wall abnormalities in *C. sinensis* pollen tubes

**Figure 5:**
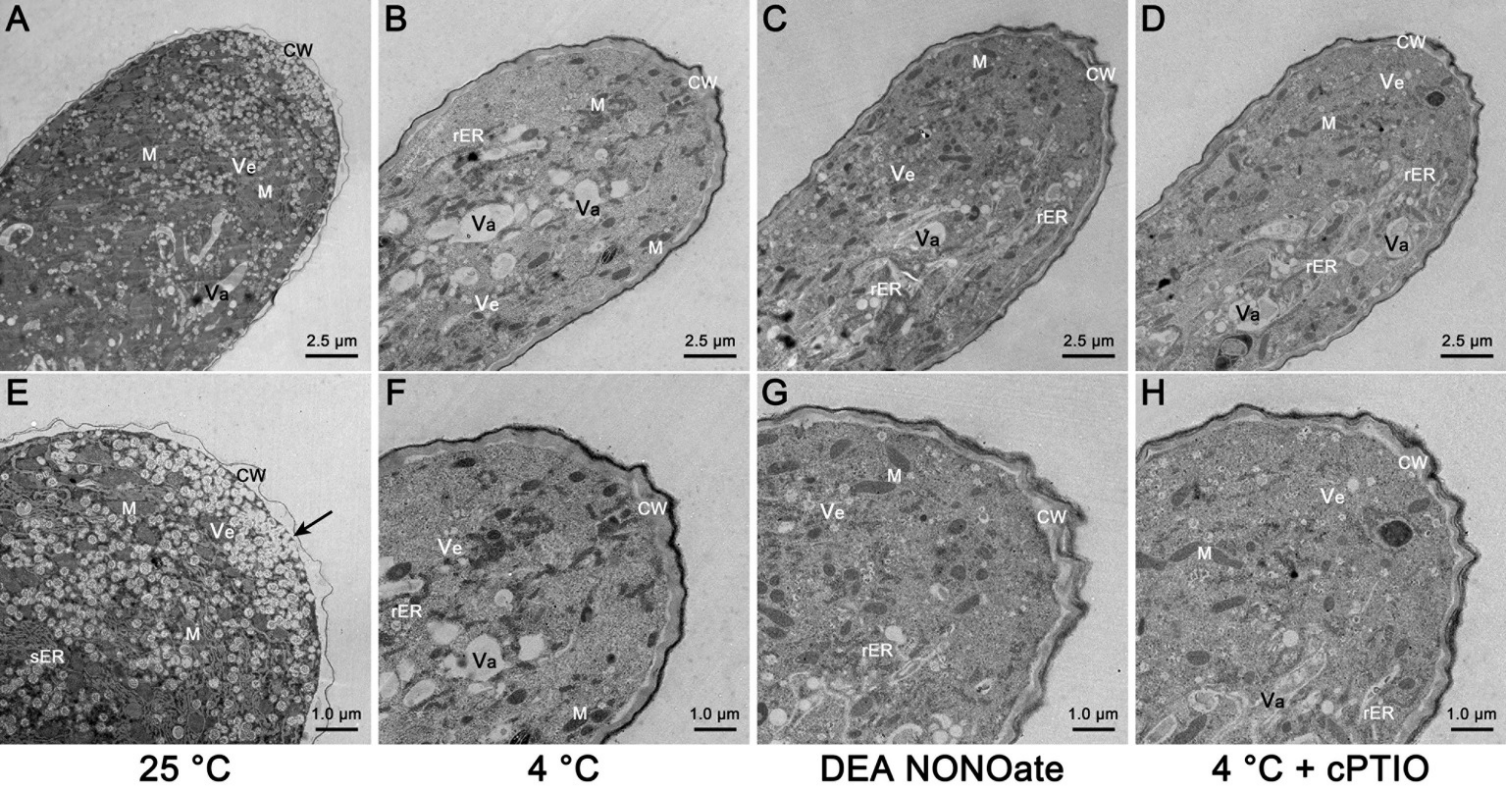
**Electron micrographs of *C. sinensis* pollen tubes after 1 h of cold stress or DEA NONOate treatment**. The extreme apical zone of the control pollen tube was filled with numerous secretory vesicles (Ve), and a large number of other organelles, particularly mitochondria (M) and smooth endoplasmic reticulum (sER), and wrapped in a thin, translucent, bumpy cell wall **(A,E)**. The black arrows indicate the fusion of vesicles with the plasma membrane **(E)**. The polarized distribution of vesicles, mitochondria and sER was disrupted, and rough endoplasmic reticulum (rER) and vacuoles (Va) existed in the tip region; the morphology of the cell wall (CW) was changed by the cold stress **(B,F)** or 25 μM DEA NONOate treatment **(C,G)**. Treatment with 200 μM cPTIO effectively reduced the effects of cold stress on the cell wall ultrastructure, but there were negligible changes in the distribution and ultrastructure of the organelles **(D,H)**. M, mitochondria; Ve, vesicle; Va, vacuole; CW, cell wall.

TEM was performed, and the results showed that the extreme apical zone of the pollen tube was filled with numerous secretory vesicles in the control condition (Figures 5A,E). Fusion of the vesicles with the plasma membrane was frequently observed, as shown by the black arrows (Figure 5E), indicating that the cell wall materials were actively released into the cell wall. A large number of other organelles, particularly mitochondria and smooth endoplasmic reticulum (sER), accumulated in the subapical zone (Figures 5A,E). However, substantial variation was observed in the pollen tube tips that were treated with cold stress (Figures 5B,F) or exogenous NO for 1 h (Figures 5C,G). The most obvious change was a disruption of the distribution of organelles, as shown by the sharp decline in the number of vesicles, mitochondria and sER, and the feature of other organelles, including the rough endoplasmic reticulum (rER) and vacuoles at the tip of the pollen tube (Figures 5B,C,F,G). Moreover, the configuration of the rER was altered, and it appeared to wrap around vacuoles and other organelles when treated with cold stress (Figure S3A) or exogenous NO (Figure S3B). Furthermore, the use of cPTIO only partly reversed the effect of cold stress on the organelle ultrastructure of the pollen tubes (Figures 5D,H), suggesting that NO is not a unique factor in the process in which cold stress affects the organelle ultrastructure of pollen tubes.

In the control pollen tubes, thick, brown, smooth cell walls were attached at the base of the pollen tube (Figures S4A,B) to maintain the mechanical support, whereas the cell wall at the tip region was thin, translucent and bumpy to maintain the high flexibility of the tip region (Figures 5A,E); these differences in the cell wall resulted in fast polarized growth of the pollen tube. However, the typical feature of the cell wall at the tip region was changed to that of a cell wall at the base region after the cold stress treatment (Figures 5B,F) or treatment with 25 μM DEA NONOate (Figures 5C,G). In addition, treatment with 200 μM cPTIO effectively reduced the effects of cold stress on the cell wall ultrastructure (Figures 5D,H), indicating that the cell wall was another key factor in the process of NO involvement in cold-inhibited *C. sinensis* pollen tube growth. Thus, we hypothesized that the cold-induced NO disrupts the organization of the cell wall, resulting in the retarded growth of the tubes and tip swelling.

### Effects of cold stress and no treatment on the distribution of pectin and AGPs

**Figure 6:**
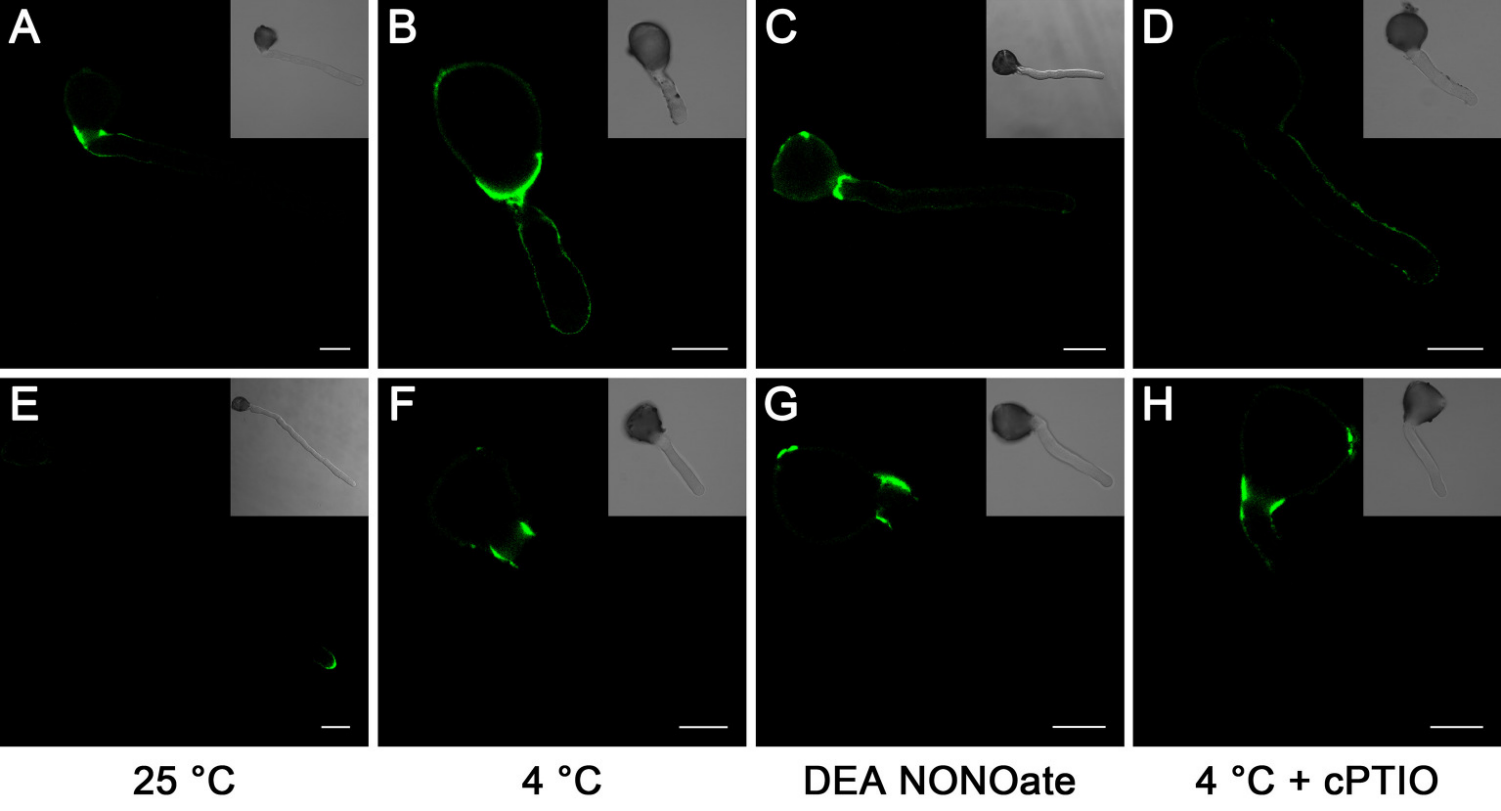
**Effects of cold stress and DEA NONOate on the distribution of acidic pectins and esterified pectins in the pollen tube cell wall of *C. sinensis***. LM19 labeling of the control pollen tubes observed by LSCM showed that strong fluorescence occurred in the basal site of the tube wall and decreased gradually toward the tip region of the pollen tube **(A)**, but fluorescence occurred along the entire pollen tube wall, including the tip region, in the pollen tubes treated with cold stress **(B)**, or 25 μM DEA NONOate **(C)**. The fluorescence signal of the LM19 labeled acidic pectins at the pollen tube tip was decreased by 200 μM cPTIO under cold stress **(D)**. LM20 labeling of the control pollen tubes observed by LSCM showed that the esterified pectins localized to the tip region of the pollen tubes **(E)**. LM20 labeling of the pollen tubes treated with cold stress or 25 μM DEA NONOate observed by LSCM showed that the esterified pectins accumulated only in the basal region near the germinating aperture **(F,G)**. Treatment with 200 μM cPTIO increased the distribution of esterified pectins on the shank of the pollen tubes but was still not detected in the pollen tube tip region **(H)**. Corresponding bright field images are shown at a reduced size. At least 20 pollen tubes were observed and photographed in each of three replicates, and one representative image per treatment was displayed. Bar = 20 μm.

**Figure 7:**
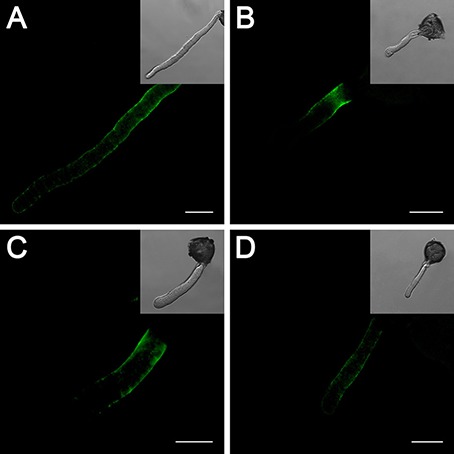
**Effects of cold stress and DEA NONOate on the distribution of AGPs in *C. sinensis* pollen tubes**. Pollen tubes incubated under standard conditions exhibited a characteristic dot-strengthening with remarkable periodicity of AGPs deposition along the entire length, as shown by immunolocalization with the LM2 antibodies, and the ring-like structures based on dot-strengthening were visualized in the apical region of the pollen tubes **(A)**. After treatment with cold stress or 25 μM DEA NONOate, the AGPs distribution showed no dot-strengthened and ring-like structures, and the fluorescence signal was deposited only on the shank region of the pollen tubes **(B,C)**. The fluorescence signal of the AGPs occurred along the entire pollen tube wall, including the tip region, under cold stress after 200 μM cPTIO treatment **(D)**. Corresponding bright field images are shown at a reduced size. At least 20 pollen tubes were observed and photographed in each of three replicates, and one representative image per treatment was displayed. Bar = 20 μm.

To further confirm the role of the cell wall, we studied the distribution of the cell wall components in pollen tubes. In pollen tubes grown under standard conditions, the localization of LM19-reactive pectin indicates the distribution of acidic pectin only in the basal regions of the tube, and the intensity of the antigen signal decreases gradually toward the apex of the tube (Figure 6A), whereas the localization of the LM20-reactive esterified pectin was limited to the very tip of the growing tubes (Figure 6E). In contrast, pollen tubes treated with cold stress (Figures 6B,F) or 25 μM DEA NONOate (Figures 6C,G) showed completely different pectin distributions compared with the control pollen tubes (Figures 6A,E); for example, acidic pectins were detected across the entire surface of the pollen tubes, including the tips (Figures 6B,C), and esterified pectins were detected only in the basal region near the germinating aperture (Figures 6F,G). Notably, the fluorescence signal of acidic pectin at the pollen tube tip was decreased by 200 μM cPTIO under cold stress (Figure 6D). In addition, treatment with cPTIO increased the distribution of esterified pectin on the shank of the pollen tubes, although the signal was still not detected at the pollen tube tip (Figure 6H). Furthermore, pollen tubes grown under standard conditions showed a characteristic dot-strengthening with remarkable periodicity of AGPs deposition along the entire length, as revealed by immune-localization with the LM2 antibodies, and the ring-like structures based on dot-strengthening were visualized, particularly in the apical region of the pollen tubes (Figure 7A) In contrast, the pollen tubes that were treated with cold stress or 25 μM DEA NONOate showed a completely different distribution of AGPs compared to the control. The dot-strengthening feature and the ring-like structures at the tip disappeared, and the fluorescence signal was observed only on the shank region of the pollen tubes (Figures 7B,C). However, the changes in the AGPs distribution caused by cold stress were effectively reversed by 200 μM cPTIO (Figure 7D).

### Signaling pathway related DEGs quantified and identified from cold stress or no treatments

**Figure 8:**
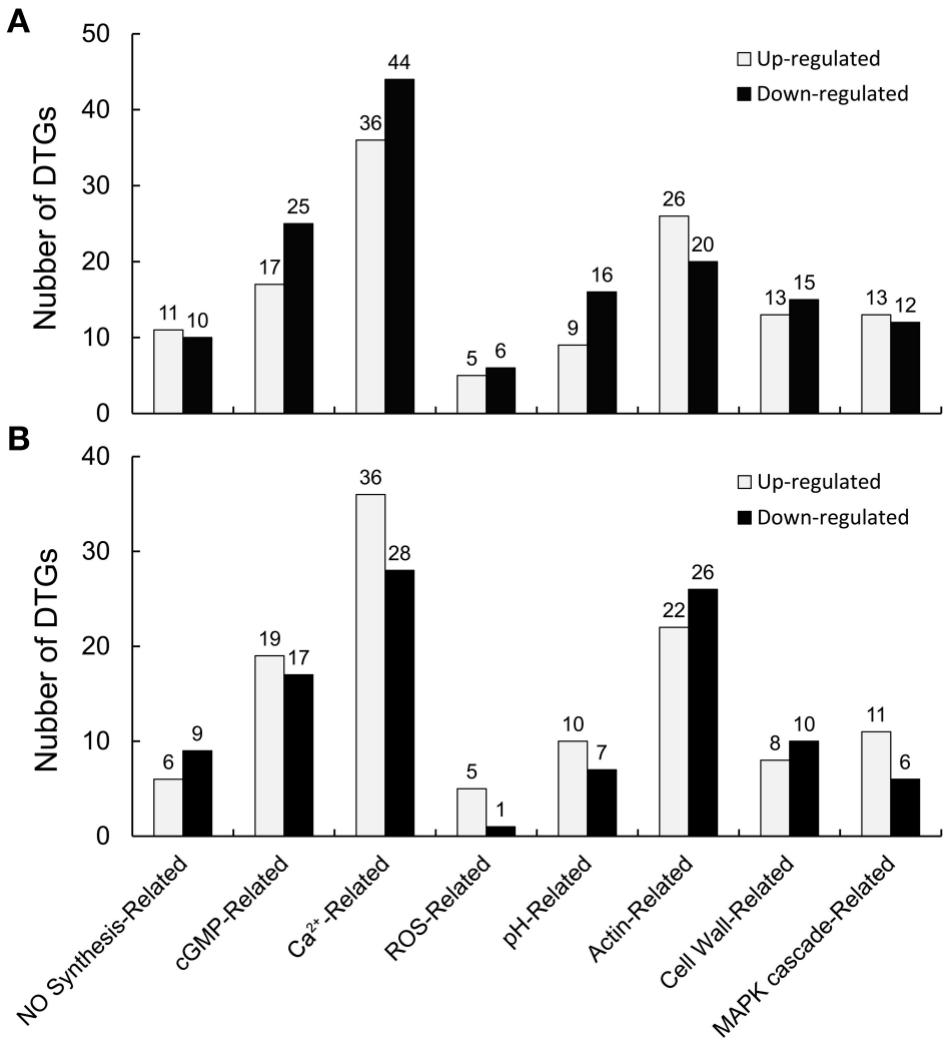
**Signaling pathway-related differentially expressed genes (DEGs) were identified after the cold stress or DEA NONOate treatments**. The transcriptomic analysis identified 278 signaling pathway-related DEGs (130 up-regulated genes and 148 down-regulated genes, 130/148) from the pollen tubes treated with cold stress **(A)** and 221 signaling pathway-related DEGs (117/104) from pollen tubes treated with 25 μM DEA NONOate **(B)**, involving the NO synthesis, cGMP, Ca^2+^, ROS, pH, actin, the cell wall, and the MAPK cascade signal pathways.

Differentially expressed genes (DEGs) were identified according to Zhao et al. (2015), with a probability >0.7 and an estimated absolute |log_2_Ratio| ≥ 1.0. Comparing the CK library (CK1, CK2, and CK3) with the LT library (LT1, LT2, and LT3), scilicet CK-VS-LT, there were 278 signaling pathway-related DEGs (130 genes up-regulated and 148 genes down-regulated, 130/148), among which there were 12 genes associated with NO synthesis and 42, 80, 11, 25, 46, 28, and 25 genes related to cGMP, Ca^2+^, ROS, pH, actin, the cell wall, and the MAPK cascade, respectively (Figure 8A, Table S2). Similarly, 221 signaling pathway-related DEGs (117/104) were detected in CK-VS-NO, and the numbers of DEGs involved in NO synthesis, cGMP, Ca^2+^, ROS, pH, actin, the cell wall, and the MAPK cascade were as follows: 15, 36, 64, 6, 17, 48, 18, and 17, respectively (Figure 8B, Table S3). In addition, more DEGs were involved in Ca^2+^ signaling than other signaling-related DEGs that were identified in the treatments, indicating the importance of Ca^2+^ in the process of the pollen tube response to cold stress and NO treatments.

In this study, hundreds of signaling pathway-related DEGs involved in the signal transduction pathways responding to cold stress (CK-VS-LT) and exogenous NO treatments (CK-VS-NO) were identified, and 89 genes were co-expressed in CK-VS-LT and CK-VS-NO (Figure S5A), including 5 NO synthesis-related genes, 16 cGMP-related genes, 21 Ca^2+^-related genes, 3 ROS-related genes, 9 pH-related genes, 20 actin-related genes, 7 cell wall-related genes, and 8 MAPK cascade-related genes (Table 1). Among these genes, some genes that were closely related to pollen tube polarized growth were detected, such as cyclic nucleotide-gated ion channels (CNGCs, Unigene4594_All and Unigene8255_All), Rac-like GTP-binding protein (RACs, CL1173.Contig1_All, and Unigene4635_All), actin-depolymerizing factor (ADF, Unigene20449_All), callose synthase (Unigene17733_All), pectin methyl esterase (PME, Unigene4524_All and Unigene12294_All), and glutamate receptor (GLRs, CL4694.Contig1_All, and CL6126.Contig4_All). Interestingly, Ca^2+^ signaling related genes were the most common, and they accounted for 23.60% of all co-expressed genes (Figure S5B), which further confirmed that Ca^2+^ plays an important role in the process of the pollen tube response to cold stress.

**Table 1:** **Co-expressed DEGs in CK-VS-LT and CK-VS-NO involved in NO signaling pathway under cold stress**.

**Classification**	**Gene ID**	**log_2_ ratio (LT/CK)**	**log_2_ ratio (NO/CK)**	**Up/Down regulation**	**Function annotation**
NO synthesis-related	Unigene20092_All	2.08	1.60	Up	Cytochrome P450 89A2
	CL632.Contig6_All	1.68	1.07	Up	Spermine synthase
	Unigene20533_All	1.59	1.61	Up	Cytochrome P450 51G1
	Unigene1619_All	−1.45	−1.63	Down	Cytochrome P450 98A1
	Unigene14044_All	−1.84	−1.77	Down	Cytochrome P450 707A3
cGMP-related	CL4986.Contig3_All	4.32	2.05	Up	Protein-tyrosine-phosphatase
	Unigene4594_All	3.56	2.13	Up	Cyclic nucleotide-gated ion channel 2, CNGC2
	CL3140.Contig3_All	2.51	2.03	Up	Protein tyrosine phosphatase
	Unigene8255_All	1.83	1.38	Up	Cyclic nucleotide-gated ion channel 1, CNGC1
	CL6165.Contig14_All	1.67	1.79	Up	Protein tyrosine kinase
	Unigene14080_All	1.60	1.77	Up	Proline-rich receptor-like protein kinase 14
	CL1828.Contig1_All	1.53	2.27	Up	Protein tyrosine kinase
	CL35.Contig18_All	1.51	1.06	Up	Protein serine/threonine/tyrosine kinase
	CL33.Contig1_All	1.31	1.39	Up	Protein tyrosine kinase
	CL6211.Contig8_All	1.21	1.32	Up	Protein tyrosine kinase
	CL2521.Contig1_All	−1.11	−1.11	Down	Protein tyrosine kinase
	Unigene12095_All	−1.45	−1.38	Down	LRR receptor-like serine/threonine-protein kinase
	Unigene22328_All	−1.49	−1.02	Down	Cyclic nucleotide-gated ion channel 6, CNGC6
	Unigene1890_All	−1.65	−1.34	Down	Transmembrane receptor protein tyrosine kinase
	Unigene19763_All	−2.23	−2.46	Down	Protein tyrosine kinase
	CL1713.Contig1_All	−2.83	−2.02	Down	Non-membrane spanning protein tyrosine kinase
Ca^2+^-related	CL2086.Contig1_All	3.53	2.92	Up	CBL-interacting serine/threonine-protein kinase 14,CIPK14
	CL1369.Contig1_All	3.51	3.57	Up	Calcium-dependent protein kinase 5, CDPK5
	CL1173.Contig1_All	2.51	2.51	Up	Rac-like GTP-binding protein 1, RAC1
	CL421.Contig4_All	2.46	2.06	Up	Calcium-binding protein CML
	Unigene12307_All	2.13	1.44	Up	Calcineurin B-like protein 3, CBL3
	Unigene12051_All	2.00	3.12	Up	Calcium-binding protein CML38
	Unigene20272_All	1.81	2.14	Up	Calcium-transporting ATPase 2
	CL1460.Contig3_All	1.80	2.29	Up	Ca^2+^-binding
	Unigene12152_All	1.48	1.98	Up	Calcium-transporting ATPase 3
	CL6126.Contig4_All	1.42	1.05	Up	Glutamate receptor 2.7-like, GLR2.7
	Unigene3698_All	1.25	1.05	Up	Calmodulin 2, CaM2
	CL2404.Contig1_All	1.21	2.16	Up	Calcium-transporting ATPase 12
	Unigene17839_All	1.20	2.12	Up	Calcium-transporting ATPase 4
	Unigene1219_All	1.01	1.32	Up	Two pore calcium channel protein 1-like, TPC1
	Unigene22270_All	−1.03	−1.23	Down	Calcineurin B-like protein, CBL1
	CL1248.Contig4_All	−1.62	−1.41	Down	Calcium-dependent protein kinase 17, CDPK17
	Unigene2215_All	−1.99	−1.17	Down	Calmodulin-binding transcription activator 3
	CL4694.Contig1_All	−2.12	−2.86	Down	Glutamate receptor 2.2-like, GLR2.2
	Unigene4635_All	−2.47	−2.36	Down	Rac-like GTP-binding protein 5, RAC5
	CL3601.Contig1_All	−2.50	−2.08	Down	Ca^2+^-binding
	CL4023.Contig1_All	−3.70	−3.70	Down	Calmodulin-binding transcription activator 4
ROS-related	CL5149.Contig11_All	3.35	1.65	Up	Peroxidase
	Unigene10633_All	3.13	2.12	Up	NADPH oxidase, NOX
	Unigene19984_All	1.47	1.57	Up	α-dioxygenase 1, NAD(P)H oxidase activity
pH-related	CL6038.Contig1_All	6.39	7.22	Up	Plasma membrane H^+^-ATPase, PM H^+^-ATPase
	Unigene11857_All	6.28	6.81	Up	Na^+^/H^+^ antiporter
	CL119.Contig5_All	2.26	2.26	Up	V-type proton ATPase catalytic subunit A
	Unigene6325_All	1.96	1.28	Up	Cation/H^+^ antiporter 15
	CL287.Contig3_All	1.36	1.28	Up	Cation/H^+^ antiporter 15
	CL853.Contig4_All	1.26	1.01	Up	Inorganic pyrophosphatase
	CL402.Contig21_All	1.09	1.46	Up	Cation/H^+^ antiporter 15
	CL332.Contig46_All	−1.50	−1.50	Down	Vacuolar proton translocating ATPase 100 kDa subunit
	Unigene8798_All	−2.48	−1.13	Down	Cation/H^+^ antiporter 15-like
Actin-related	Unigene15928_All	5.18	6.72	Up	Actin
	Unigene6231_All	4.55	6.95	Up	Actin-binding
	CL2575.Contig3_All	3.92	2.48	Up	Formin-like protein 3
	Unigene3512_All	3.67	3.21	Up	Myosin-H heavy chain-like
	Unigene20449_All	2.78	2.86	Up	Actin-depolymerizing factor 1, ADF1
	Unigene20955_All	2.27	2.36	Up	Actin cytoskeleton organization
	CL1355.Contig4_All	1.95	2.84	Up	Myosin-J heavy chain-like
	CL3551.Contig2_All	1.84	2.47	Up	Kinesin-1
	Unigene1957_All	1.83	1.86	Up	Kinesin family member 2/24
	CL274.Contig4_All	1.72	1.43	Up	Caltractin (Ca^2+^-binding protein)
	Unigene2298_All	1.30	1.34	Up	Myosin-Vb-like
	Unigene19227_All	1.07	1.01	Up	Actin nucleation
	CL4715.Contig1_All	1.00	1.91	Up	Positive regulation of actin nucleation
	Unigene15770_All	−1.06	−1.01	Down	Actin-related protein 4
	Unigene8236_All	−1.12	−1.43	Down	65-kDa microtubule-associated protein 3
	Unigene22115_All	−1.13	−1.35	Down	Villin-1
	Unigene22529_All	−1.55	−2.94	Down	Formin-like protein 7
	CL3197.Contig7_All	−1.58	−2.30	Down	Formin-like protein 20
	Unigene2909_All	−2.78	−1.19	Down	Myosin-Vb-like
	CL797.Contig2_All	−4.13	−2.00	Down	F-actin-capping protein subunit α
Cell wall-related	CL5329.Contig2_All	2.31	1.96	Up	β-1,3-galactosyltransferase 20
	Unigene4524_All	2.12	1.68	Up	Pectin methyl esterase 2, PME2
	Unigene17733_All	2.04	1.09	Up	Callose synthase 12
	Unigene12294_All	1.82	1.74	Up	Pectin methyl esterase 1, PME1
	CL240.Contig9_All	−1.33	−2.53	Down	Pollen Ole e1 allergen and extensin family protein
	Unigene11072_All	−1.50	−1.35	Down	Arabinogalactan peptide 22, AGP22
	CL1401.Contig2_All	−2.65	−1.18	Down	Probable pectinesterase/pectinesterase inhibitor 21
MAPK cascade-related	Unigene20102_All	1.87	1.05	Up	Mitogen-activated protein kinase kinase kinase 3, MAP3K3
	Unigene3464_All	1.50	1.69	Up	Activation of MAPKK activity
	Unigene18265_All	1.50	1.49	Up	WRKY transcription factor 21
	Unigene4667_All	1.43	1.04	Up	Mitogen-activated protein kinase kinase kinase, MAP3K
	CL580.Contig48_All	1.27	1.07	Up	Protein kinase and PP2C-like domain-containing protein
	Unigene18965_All	1.24	1.32	Up	Probable CCR4-associated factor 1 homolog 11
	Unigene21639_All	−1.27	−1.83	Down	WRKY transcription factor 19
	Unigene7269_All	−1.60	−1.05	Down	WRKY transcription factor 7

### Quantitative real-time PCR (qRT-PCR) analysis

**Figure 9:**
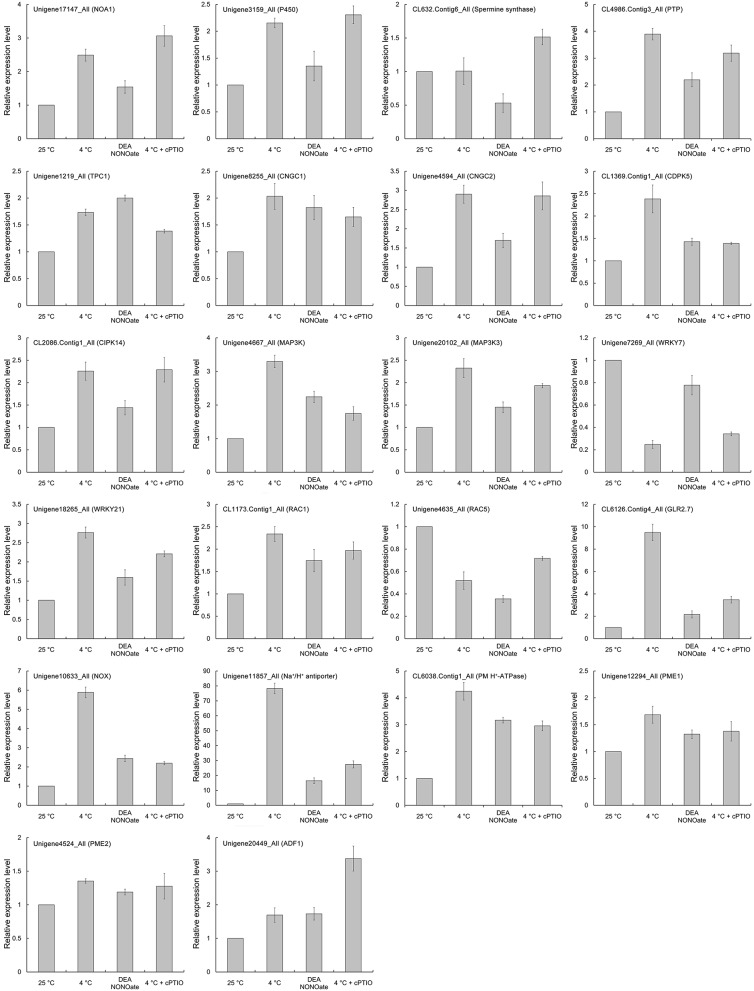
**QRT-PCR validation for 22 co-expressed DEGs in CK-VS-LT and CK-VS-NO**.

To validate the expression profiles of the genes in our Illumina RNA-Seq results and to further verify the key functions of NO in the pollen tube response to cold stress, the expression levels of 22 critical DEGs were analyzed using qRT-PCR after treatment with cold stress (4°C), 25 μM DEA NONOate or 4°C and 200 μM cPTIO. These genes encode the following: NO-associated protein 1 (NOA1), spermine synthase, cytochrome P450 93A1-like (P450), protein tyrosine phosphatase (PTP), two pore calcium channel protein 1-like (TPC1), cyclic nucleotide-gated ion channels (CNGCs), calcium-dependent protein kinase SK5 (CDPK5), mitogen-activated protein kinase kinase kinase (MAP3K), WRKY transcription factors (WRKYs), Rac-like GTP-binding proteins (RACs), glutamate receptor 2.7-like (GLR2.7), NADPH oxidase (NOX), Na^+^/H^+^ antiporter, plasma membrane H^+^-ATPase (PM H^+^-ATPase), PME, and actin-depolymerizing factor 1 (ADF1). As shown in Figure 9, the expression tendency of these genes exhibited a close similarity to the Illumina RNA-Seq results (Table 1). In addition, the changes in the expression levels of most genes were relieved by 200 μM cPTIO treatment under cold stress, such as PTP, TPC1, CNGC1, CDPK5, RACs, GLR2.7, NOX, Na^+^/H^+^ antiporter, PME1, and ADF1, indicating that these genes participated in the pollen tube response to cold stress, possibly through the regulation of the NO signaling pathway.

**Figure 10:**
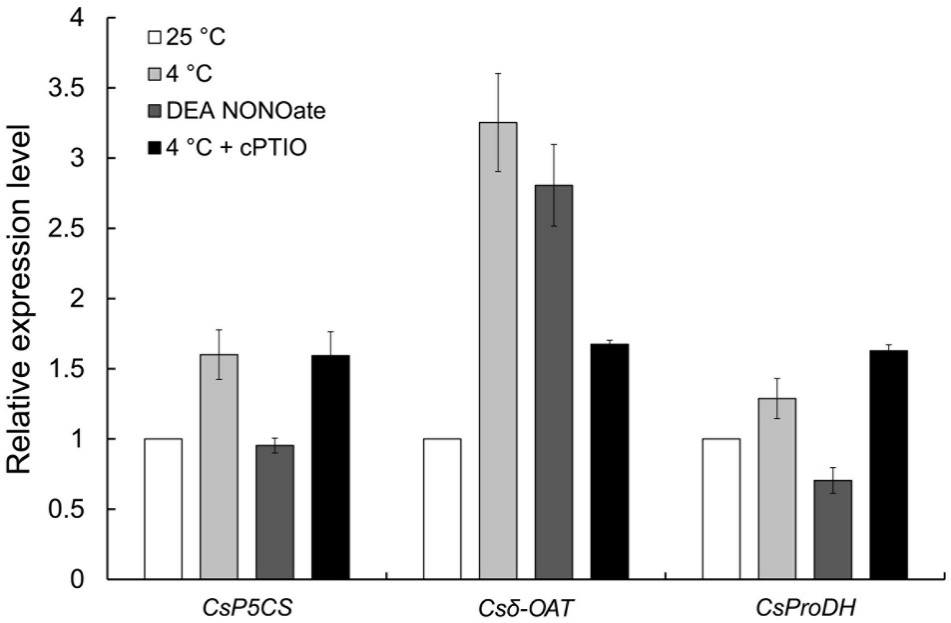
**The expression levels of three rate-limiting enzyme genes in Pro metabolism were analyzed by qRT-PCR**. Cold-induced NO promotes the expression of *Cs*δ*-OAT* and inhibits the expression of *CsProDH* but does not affect the expression of *CsP5CS*.

In our previous study, proline (Pro) accumulation played an important role in the process of NO involvement in cold-inhibited *C. sinensis* pollen tube growth (Wang et al., 2012), but the mechanism of Pro accumulation remains unclear. Here, the expression of three rate-limiting enzyme genes in Pro metabolism, including Δ^1^-pyrroline-5-carboxylate synthetase (*P5CS*), ornithine-δ-aminotransferase (δ*-OAT*) and proline dehydrogenase (*ProDH*), were detected by qRT-PCR (Figure 10). The results showed that the expression of *CsP5CS* increased after cold stress treatment but did not change after treatment with 25 μM DEA NONOate. Additionally, the expression of *Cs*δ*-OAT* was induced by cold stress or exogenous NO, and the induction that resulted from cold stress was significantly reduced by 200 μM cPTIO. Interestingly, exogenous NO decreased the expression of *CsProDH*, and cPTIO caused a greater increase in *CsProDH* expression under cold stress compared with cold stress-treated pollen tubes. These results suggest that NO regulated Pro accumulation by increasing the expression of *Cs*δ*-OAT* instead of *CsP5CS* and by reducing the expression of *CsProDH* in *C. sinensis* pollen tubes responding to cold stress.

## Discussion

Research over the last few decades has identified NO as an important signaling molecule with diverse biological functions in plants. NO plays a crucial role in growth and development, from germination to senescence, and is also involved in plant responses to biotic and abiotic stresses, including cold stress (Sehrawat et al., 2013b). In addition, previous investigations have demonstrated that NO is involved in the regulation of pollen tube growth, particularly in the polarized tip (Prado et al., 2004). Our data show that both NO and cold stress inhibit *C. sinensis* pollen tube growth and lead to tip morphological abnormalities and that the NO scavenger cPTIO is able to effectively mitigate the effects of cold stress on pollen tubes, implying that NO participates in the process of cold stress-inhibited *C. sinensis* pollen tube growth (Wang et al., 2012). This is consistent with the results of (Prado et al., 2004, 2008) who reported NO as a negative regulator of pollen tube growth in *Lilium longiorum* and *Arabidopsis thaliana*.

It is well known that NO production is mainly mediated through three NO synthases (NOS) with different localizations and functions in animals, which catalyze the conversion of L-Arg to L-citrulline and NO (Qiao and Fan, 2008). However, the pathways for producing NO in plant tissues are complicated, diverse and undefined and remain a matter of discussion. Current studies have revealed that NOS activity has also been detected in higher plants, although no direct gene coding for a canonical NOS protein has been found in the genomes of *Arabidopsis* or any other higher plants (Domingos et al., 2015). For example, Zhao et al. (2007) reported that NOS-dependent NO production was associated with salt tolerance in *Arabidopsis*, and NOS-like activity-dependent endogenous NO production enhanced the tolerance to cold stress in *Chorispora bungeana* suspension culture cells (Liu et al., 2010). In our previous report, NOS-like activity was confirmed to participate in the cold-induced NO production in *C. sinensis* pollen tubes (Wang et al., 2012). In the present study, the expression of the NO-associated protein 1 gene (*NOA1*, Unigene17147_All) was induced by cold stress, and cPTIO increased the effects of cold stress (Figure 9), which further supports the conclusion about cold stress-induced NO production partly from NOS-like activity in our previous studies. In addition to NOS-mediated NO production, several other NO biosynthetic enzymes may function in plant cells, including the NAD(P)H-dependent nitrate reductase (NR), xanthine oxidase/dehydrogenase and cytochrome P450 (Zhao et al., 2009; Sanz et al., 2015). Many potential NO production-related genes were also identified by transcriptomic analyses in the process of the *C. sinensis* pollen tube response to cold stress, such as the cytochrome P450 family genes and arginine metabolism-related genes (Table S3). Interestingly, NR genes were not among the differentially expressed genes after treatment with cold stress, although the role of NR in the cold-induced NO accumulation in *C. sinensis* pollen tubes cannot be excluded. These results indicate that the sources of NO production under cold stress may be the result of the synergism of several pathways in *C. sinensis* pollen tubes.

As the most ubiquitous second messenger, Ca^2+^ dependent signaling networks can respond to many physiological processes in plant cells, and it has been shown that there is a close coupling between the intracellular tip-focused Ca^2+^ gradient and the polarized growth of the pollen tube (Holdaway-Clarke and Hepler, 2003). Currently, increasing evidence confirms that there is complicated crosstalk among NO and Ca^2+^ signaling pathways. Prado et al. (2004) reported that a putative NO-cGMP signaling pathway induced pollen tube reorientation through effects on cytoplasmic Ca^2+^ concentrations in lily. Similarly, NO was also found to modulate the cytoplasmic Ca^2+^ gradient to regulate *Pinus bungeana* pollen tube development largely by mediating Ca^2+^ influx, which is most likely dependent on cGMP-activated channels in pollen tubes (Wang et al., 2009). In the present study, we found that the Ca^2+^ gradient was disrupted by both cold stress and NO, and the disruption was relieved by cPTIO. Combined with our previous reports (Wang et al., 2012), it is reasonable to speculate that cold-induced NO inhibits the polarized growth of *C. sinensis* pollen tubes dependent on the damage to the cytoplasmic Ca^2+^ gradient at the pollen tube tip zone. Additionally, pharmacological and biochemical studies have shown that NO signaling in plants is mediated by cGMP (Prado et al., 2004) and cyclic nucleotide-gated ion channels (CNGCs), which are permeable to both monovalent and divalent cations (typically K^+^, Na^+^, and Ca^2+^) and are activated by cGMP and/or cAMP (Wang et al., 2013). Our data indicate that the expression of multiple CNGC genes is up-regulated after NO or cold stress treatment and that the expression of a large number of genes that encode proteins that activate CNGCs is induced, such as protein tyrosine kinases (PTKs) and protein tyrosine phosphatases (PTPs), which regulate the sensitivity of CNGCs on cGMP by catalytic phosphorylation (Chae et al., 2007). Recently, glutamate receptor-like channels (GLRs), a putative group of pollen Ca^2+^ channels, were identified, and their Ca^2+^ transport activities in pollen tubes have been confirmed based on direct electrophysiological, pharmacological and genetic evidence (Konrad et al., 2011; Michard et al., 2011). Interestingly, two GLR genes were identified from DEGs of CK-Vs-LT and CK-Vs-NO, implying that GRLs participate in the process in which NO results in a Ca^2+^ gradient disruption at the pollen tube tip after treatment with cold stress. Furthermore, the expression of another class of Ca^2+^ channel protein family genes, two pore calcium channel protein genes (TPC, Unigene1219_All), was shown to be up-regulated after NO or cold stress treatment, and the induction of cold stress was inhibited by cPTIO, indicating that TPC was also involved in the regulatory process of cold-induced NO disruption of the Ca^2+^ gradient at the pollen tube tip. These data suggest that NO regulates the cytoplasmic Ca^2+^ gradient largely by mediating Ca^2+^ fluxes under cold stress, which is most likely dependent on various Ca^2+^ channels in *C. sinensis* pollen tubes, such as CNGCs, GLRs, and TPCs.

The downstream proteins that can bind Ca^2+^ and act upon changes in Ca^2+^ concentrations to perform specific functions play important roles in the process of pollen tube tip growth (Konrad et al., 2011), particularly Ca^2+^ sensor and relay proteins, including calmodulin (CaM), CaM-like proteins (CMLs), Ca^2+^-dependent protein kinases (CDPKs), calcineurin B-like proteins (CBLs), and CBL-interacting protein kinases (CIPKs; Gutermuth et al., 2013; Steinhorst and Kudla, 2013; Zhou et al., 2014). Recently, Zhou et al. (2015) reported that *Arabidopsis* CIPK19 is required for pollen tube growth and polarity by participating in Ca^2+^ homeostasis dependent on the modulation of Ca^2+^ influx. In our investigation, we also found that multiple sensors and relay protein genes were up/down-regulated in *C. sinensis* pollen tubes in response to NO or cold stress treatment. Combined with previous results, we infer that Ca^2+^ binging proteins are involved in cold-induced NO-inhibited pollen tube tip growth, which is dependent on the regulation of NO on the cytoplasmic Ca^2+^ gradient (Domingos et al., 2015). In addition, accumulating evidence indicates that cytoskeleton elements (actin/myosin cables) control cytoplasmic streaming, the distribution of the endoplasmic reticulum (ER) and the transport of secretory vesicles and that actin polymerization itself also contributes to pollen tube growth (Chen et al., 2007). Concurrently, the dynamic state of the cytoskeleton elements is controlled via numerous regulatory factors, including several actin-binding proteins activated in response to Ca^2+^ (Cárdenas et al., 2008). Moreover, Wang et al. (2009) reported that F-actin organization in the tip region of pollen tubes sensitive to NO is partly dependent on the Ca^2+^ gradient during NO signaling in *P. bungeana* pollen tubes. Our results show that cold-induced NO stimulation caused a sharp decline in the number of vesicles and ER distribution abnormality in the *C. sinensis* pollen tube tip region accompanied by a sharper Ca^2+^ gradient disruption. In addition, a large number of cytoskeleton-related genes were up/down-regulated in this process, such as actin, formin-like protein genes, myosin-like genes, kinesin genes, actin-depolymerizing factor, and the caltractin gene, which encodes a type of Ca^2+^ binding protein. This suggests that vesicular trafficking and ER distribution were perturbed by the cold-induced NO accumulation, which may be partly dependent on the regulation of the NO-induced Ca^2+^ gradient change in cytoskeleton elements, particularly the dynamic polymerization status of F-actin (Chen et al., 2009). Furthermore, mitogen-activated protein kinases (MAPKs), which are another type of important downstream target of the Ca^2+^ signal, have been confirmed to mediate the guidance response in pollen tubes (Guan et al., 2014). In addition, accumulating evidence indicates that MAPKs function as intracellular targets for NO and participate in the developmental processes of plants, including pollen tube tip growth (Arasimowicz and Floryszak-Wieczorek, 2007; Domingos et al., 2015; Sanz et al., 2015). As expected, some MAPK genes and downstream genes were induced by cold-dependent NO in *C. sinensis* pollen tubes, which is consistent with a previous report showing that NO activates a potential MAPK during NO-induced PCD in *A. thaliana* suspension cultures (Clarke et al., 2000).

It is generally accepted that there is crosstalk among NO, Ca^2+^, and ROS signaling pathways in plants, particularly in the process of pollen tube growth and the response to environmental stress (Domingos et al., 2015). For example, NADPH oxidases (NOX) are expressed in pollen tubes and localize to the plasma membrane (PM) where they produce ROS, namely H_2_O_2_, which promotes NO synthesis through NR and/or NOA1. In contrast, NO activates protein kinases (PK), enabling NOX to bind Ca^2+^, triggering more ROS production, and NO-dependent cGMP activates Ca^2+^ channels at the PM to provide adequate Ca^2+^ for NOX (Liu et al., 2009; Wilkins et al., 2011; Wudick and Feijó, 2014; Domingos et al., 2015). In addition, the activation of PM NOX also can be triggered by the CDPK in a Ca^2+^-dependent manner to produce ROS in pollen tubes (Kobayashi et al., 2007; Potocký et al., 2007; Kaya et al., 2014). Our results show that NO increases the accumulation of ROS accompanied by a significantly up-regulated expression of the NOX gene and Ca^2+^ gradient disruption in the *C. sinensis* pollen tube in response to cold stress. Regardless of the reactivity of ROS and NO, our results suggest that cold-induced NO regulates the production of ROS partly through the activation of PM NOX triggered by Ca^2+^, which further supports the results of previous studies (Kaya et al., 2014; Domingos et al., 2015). Furthermore, increasing evidence suggests that small GTPases in plants called RAC/ROPs (RACs are used in this study) function as molecular switches in the polarized cell growth of pollen tubes and root hairs and in defense-related responses (Zou et al., 2011; Huang et al., 2014). Kost et al. (1999) reported that the overexpression of *RAC1* enlarged the apical regions of the PM containing RAC1, which is correlated with the severity of the depolarized growth of pollen tubes. In the present study, the up-regulation of *RAC1* and the down-regulation of *RAC5* were detected after treatment with NO or cold stress, and the effects of cold stress were relieved by cPTIO, implying that RACs play important roles in cold-induced NO-inhibited tip growth in *C. sinensis* pollen tubes, which supports the above results of Kost et al. (1999). In addition, recent studies have shown that RACs affect the actin dynamics mediated by Ca^2+^ and/or ADF, thereby controlling the polarized growth of pollen tubes (Chen et al., 2003; Fu, 2010; Wu et al., 2011). Moreover, RACs are found to be involved in stimulating the ROS accumulation in the polarized growth of pollen tubes (Potocký et al., 2012; Kaya et al., 2014). Given the crosstalk among NO, Ca^2+^ and the ROS signaling pathway, we speculate that RACs are involved in the regulation of NO on Ca^2+^ and the ROS signaling pathway in the process of cold stress inhibiting the *C. sinensis* pollen tube tip growth.

Recently, Wilkins et al. (2015) reported that cytosolic pH ([pH]_*cyt*_) acidification was necessary and sufficient for triggering several key hallmark features of the self-incompatibility-induced PCD signaling pathway. Our data also reveal that cold-induced NO inhibits the tip growth of *C. sinensis* pollen tubes accompanied by a significant [pH]_*cyt*_ acidification, which confirms the role of pH signaling in plant pollen tube growth (Michard et al., 2009). In addition, previous studies have suggested that the changes in [pH]_*cyt*_ in the pollen tube tip are dependent on H^+^ fluxes mediated by the regulation of Ca^2+^ on PM H^+^-ATPase and Na^+^/H^+^ antiporter (SOS1) activities (Certal et al., 2008; Michard et al., 2008; Guo et al., 2009). Interestingly, whereas cold-induced NO results in *C. sinensis* pollen tube tip acidification, the expression of PM H^+^-ATPase and the Na^+^/H^+^ antiporter gene are found to be significantly up-regulated, suggesting that potential H^+^ fluxes participate in the process of pollen tube tip acidification, which is similar to reports by Sun et al. (2009) that the synergistic effect between PM H^+^-ATPase and the Na^+^/H^+^ antiporter increased H^+^ influx in root protoplasts under salt stress. Of course, the contribution of Ca^2+^ cannot be neglected in the above process (Michard et al., 2009). Furthermore, pH as a regulator of PME activity was involved in the regulation of PME on pectin status and distribution at the pollen tube cell wall (Li et al., 1994). Increasing evidence confirms that the dynamic balance between cell wall extensibility and rigidity is another key factor that regulates tip growth in pollen tubes (Chen et al., 2007, 2009). In the present study, we also found that two PME genes were up-regulated after treatment with NO or cold stress. In addition, immunolabeling with LM19 and LM20 showed that cold-induced NO changed the distribution of acidic and esterified pectins at the cell wall of *C. sinensis* pollen tubes compared to that in the controls; this is similar to data showing that an increase in the degree of cell wall rigidity and a decrease in visco-elasticity influence pollen tube growth and architecture (Parre and Geitmann, 2005; Wang et al., 2009). The characteristic dot-strengthened and ring-like structures of AGPs at pollen tubes cell wall disappeared after treatment with NO or cold stress, which is similar to the results of Chen et al. (2007). In addition, ultrastructure observation showed that cold-induced NO causes cell wall thickening, smoothing and color deepening, which is likely dependent on the above changes in cell wall construction. Taking these findings together, we speculate that cold-induced NO stimulates changes in cell wall component distributions, leading to excess wall rigidity at the tip of the pollen tube, thereby inhibiting the polarized growth of the *C. sinensis* pollen tube tip, which may partly account for the synergistic effect of pH and PME mediated by NO.

Furthermore, it is well known that higher plants accumulate free Pro in response to a number of abiotic stresses, such as drought, salinity and freezing (Zhao et al., 2009). Nevertheless, only a few reports have indicated that NO and Pro cross-talk in cold acclimation and freezing tolerance. For example, Zhao et al. (2009) showed that cold-induced NO acts as a signal to evoke Pro accumulation via enhanced synthesis and reduced degradation by regulating related genes of the Pro biosynthetic pathway in Arabidopsis, which may be a function of NO in freezing tolerance. Similarly, our previous study showed that NO participated in stimulating Pro accumulation in *C. sinensis* pollen tube responses to cold stress (Wang et al., 2012). In addition, Ruan et al. (2004) reported that NO promoted the activity of P5CS1 and decreased the activity of ProDH. Interestingly, our present study showed that cold-induced NO increased the expression of *Cs*δ*-OAT* and reduced the expression of *CsProDH* but had no effect on the expression of *CsP5CS* (Figure 10). These results reveal that NO regulated Pro accumulation by increasing the expression of *Cs*δ*-OAT* instead of *CsP5CS* and by reducing the expression of *CsProDH* in *C. sinensis* pollen tubes during the response to cold stress, although the contribution of CsP5CS cannot be ignored in cold stress-induced Pro accumulation (Figure S6).

**Figure 11:**
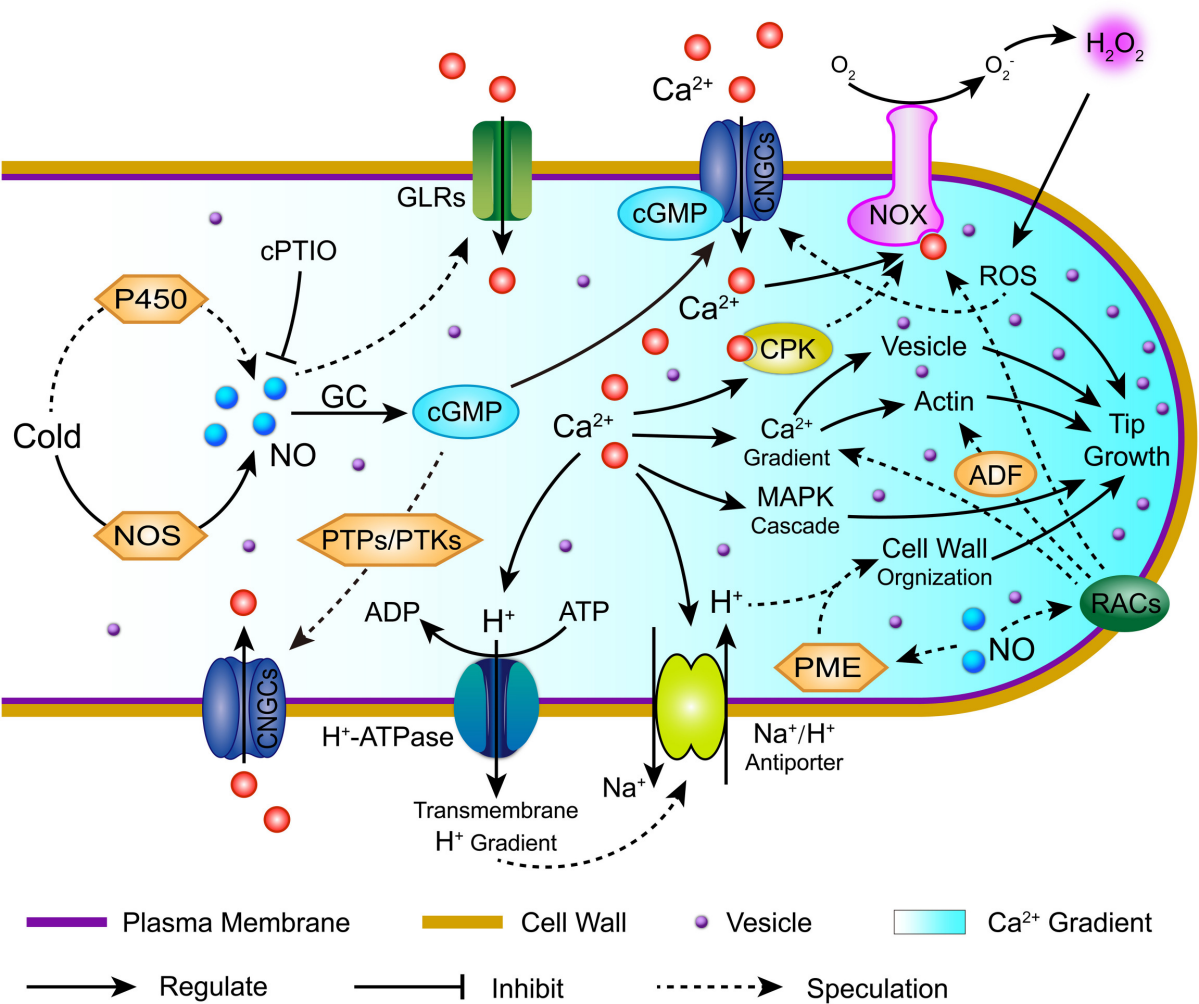
**Hypothetical model summarizing the potential signaling pathway of nitric oxide (NO) involved in cold-inhibited *C. sinensis* pollen tube growth**. This simplified model was based on the pollen tube models proposed by Wang et al. (2009 and 2012), (Wudick and Feijó, 2014), and Domingos et al. (2015). Cold stress induces an increase in NO through the synergism of several pathways in *C. sinensis* pollen tubes, such as the accumulation of NOS-like activity and cytochrome P450 activity. Consequently, the cytoplasmic Ca^2+^ gradient was regulated largely by mediating the Ca^2+^ flux, which is dependent on various Ca^2+^ channels, such as CNGCs (cGMP-activated channels), GLRs and TPCs, and this subsequently triggered secondary and tertiary regulatory networks, including Ca^2+^ sensor and relay proteins, the MAPK cascade, ROS, actin, vesicles and pH signaling. In addition, Ca^2+^-dependent [pH]_*cyt*_ acidification interacted with PMEs, leading to changes in the cell wall structure and component distribution. Furthermore, RAC signaling involved in the process of cold-induced NO inhibited *C. sinensis* pollen tube polarized growth by regulating the Ca^2+^ and ROS signaling pathways. Together, the complex signaling network dominated by NO mediates the cold-inhibited *C. sinensis* pollen tube growth. NOS, nitric oxide synthase; GC, guanylyl cyclase; PTPs, protein tyrosine phosphatases; cPTIO, 2-(4-carboxyphenyl)-4,4,5,5-tetramethylimidazoline-1-oxyl-3-oxide; PTKs, protein tyrosine kinases; CNGCs, cyclic nucleotide-gated ion channels; GLRs, glutamate receptor-like channels; CPK, calcium-dependent protein kinase; NOX, NADPH oxidase; ROS, reactive oxygen species; ADF, actin-depolymerizing factor; PME, pectin methylesterase; RACs, Rac-like GTP-binding proteins.

In summary, our cytological and transcriptomic analyses provide a more global picture of the role of NO in cold stress to inhibit the polarized tip growth of *C. sinensis* pollen tubes. A complex signaling network dominated by NO, including Ca^2+^, ROS, pH, RACs signaling, and the crosstalk among them, was investigated in the *C. sinensis* pollen tube response to cold stress, which is summarized in Figure 11. This study provided two novel findings. First, cold-induced NO causes Ca^2+^ gradient disruption in *C. sinensis* pollen tubes, most likely through Ca^2+^ fluxes mediated by various Ca^2+^ channels and through subsequently triggered secondary and tertiary regulatory networks, including Ca^2+^ sensor and relay proteins, the MAPK cascade, ROS and pH signaling. Second, [pH]_*cyt*_ acidification interacted with PMEs, leading to changes in the cell wall structure and component distributions, thereby inhibiting the polarized growth of the *C. sinensis* pollen tube tips after treatment with cold stress. Furthermore, RAC signaling is involved in the process of cold-induced NO-inhibited *C. sinensis* pollen tube polarized growth, possibly through regulating the Ca^2+^ and ROS signaling pathways, which is also an interesting result. Taken together, our study provides new insights into the multifaceted mechanistic framework for the functions of NO in cold-inhibited *C. sinensis* pollen tube growth.

## Author contributions

WW, YW, and XS designed research; WW, XS, ZS, DL, and YW performed research; WW, JP, XY, PC, and YW analyzed data; WW and YW wrote the paper; XL and YW revised this paper.

### Conflict of interest statement

The authors declare that the research was conducted in the absence of any commercial or financial relationships that could be construed as a potential conflict of interest.
