# Supplementary material for: Combined Cytological and Transcriptomic Analysis Reveals a Nitric Oxide Signaling Pathway Involved in Cold-Inhibited Camellia sinensis Pollen Tube Growth
Source: Front Plant Sci. 2016 Apr 14;7:456. doi: 10.3389/fpls.2016.00456 (PMC4830839; doi:10.3389/fpls.2016.00456)
Supplement: Supplementary file 4 [file Image1.PDF]

Figure S1

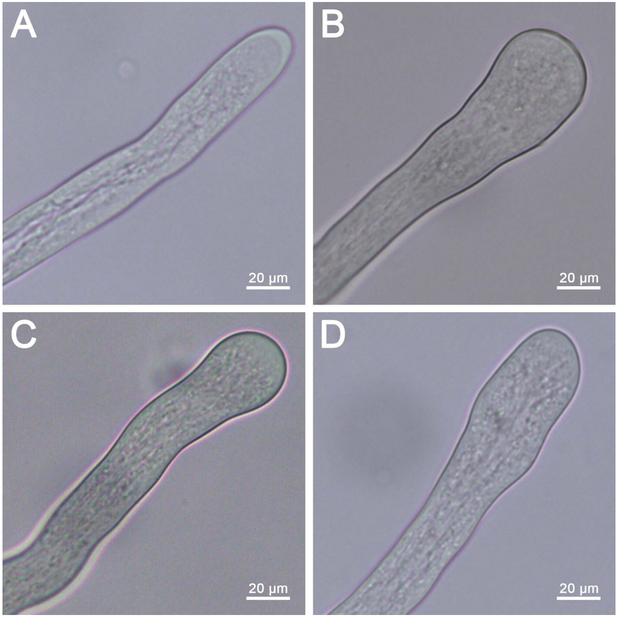

Supplemental Figure 1. Effects of cold stress or DEA NONOate on the morphology of *C. sinensis* pollen tubes after treatment for 1 h. Compared to the control (A), the pollen tubes treated with cold stress (B) or 25 μM DEA NONOate (C) exhibited swollen tips and loss of the clear zone. The effects of cold stress on the pollen tube tip morphology were reduced by 200 μM cPTIO (D).
