# Supplementary material for: Combined Cytological and Transcriptomic Analysis Reveals a Nitric Oxide Signaling Pathway Involved in Cold-Inhibited Camellia sinensis Pollen Tube Growth
Source: Front Plant Sci. 2016 Apr 14;7:456. doi: 10.3389/fpls.2016.00456 (PMC4830839; doi:10.3389/fpls.2016.00456)
Supplement: Supplementary file 5 [file Image2.PDF]

Figure S2

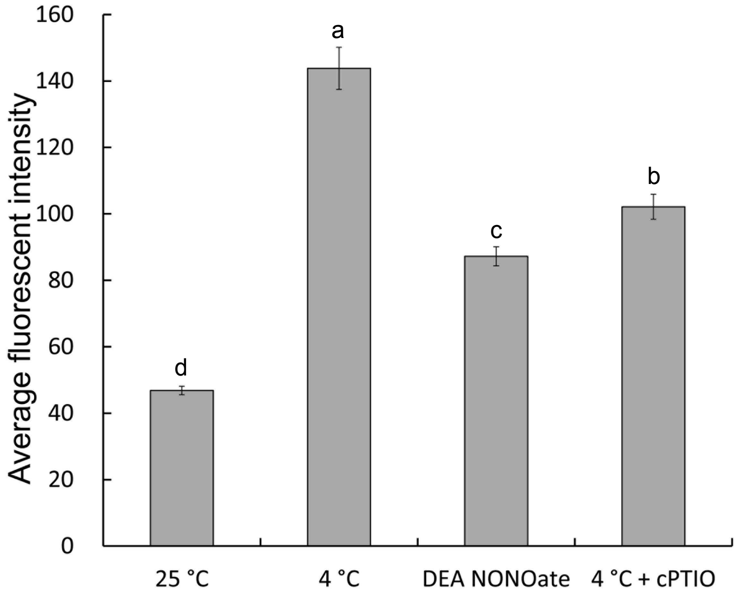

Supplemental Figure 2. Average intensity of fluorescence from the ROS specific indicator CM-H2DCF-DA in *C. sinensis* pollen tubes. Compared with the control pollen tubes, the average fluorescence intensity was significantly increased by cold stress or 25 μM DEA NONOate treatment, and the increased levels of average fluorescence intensity induced by cold stress were decreased by treatment with 200 μM cPTIO. All data are the means of three replicates ± SD (n ≥ 20).
