# Supplementary material for: Combined Cytological and Transcriptomic Analysis Reveals a Nitric Oxide Signaling Pathway Involved in Cold-Inhibited Camellia sinensis Pollen Tube Growth
Source: Front Plant Sci. 2016 Apr 14;7:456. doi: 10.3389/fpls.2016.00456 (PMC4830839; doi:10.3389/fpls.2016.00456)
Supplement: Supplementary file 6 [file Image3.PDF]

Figure S3

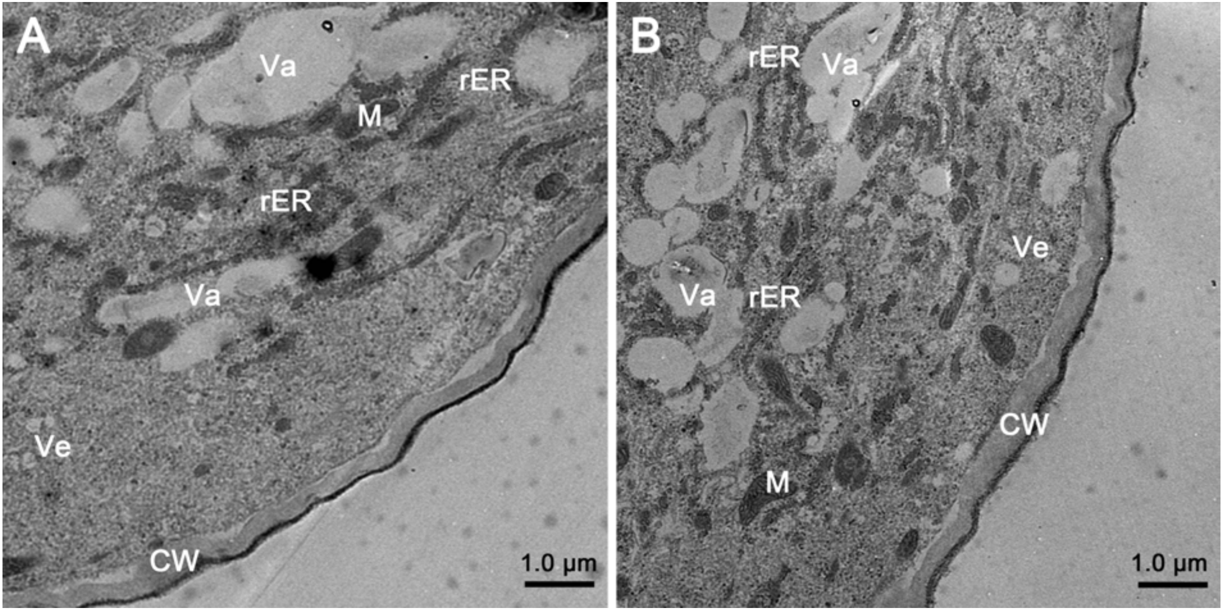

Supplemental Figure 3. Effects of cold stress or DEA NONOate on the morphology of the rER and vacuoles in *C. sinensis* pollen tubes. The configuration of the rER was altered, and it appeared to wrap around other organelles, particularly vacuoles, after the cold stress treatment (A) or 25 μM DEA NONOate (B).
