# Supplementary material for: Combined Cytological and Transcriptomic Analysis Reveals a Nitric Oxide Signaling Pathway Involved in Cold-Inhibited Camellia sinensis Pollen Tube Growth
Source: Front Plant Sci. 2016 Apr 14;7:456. doi: 10.3389/fpls.2016.00456 (PMC4830839; doi:10.3389/fpls.2016.00456)
Supplement: Supplementary file 7 [file Image4.PDF]

Figure S4

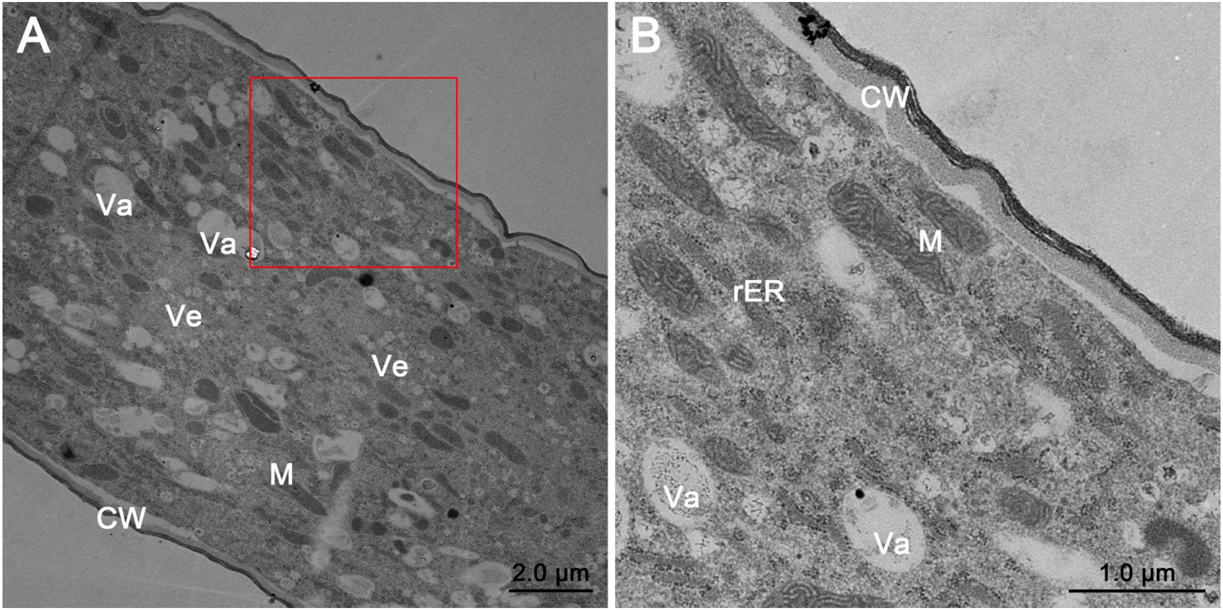

Supplemental Figure 4. Electron micrographs of *C. sinensis* pollen tube base cell walls. The thick, brown, smooth cell wall was attached at the base of the pollen tube (A). The red box region is shown at an increased size (B).
