# Supplementary material for: Combined Cytological and Transcriptomic Analysis Reveals a Nitric Oxide Signaling Pathway Involved in Cold-Inhibited Camellia sinensis Pollen Tube Growth
Source: Front Plant Sci. 2016 Apr 14;7:456. doi: 10.3389/fpls.2016.00456 (PMC4830839; doi:10.3389/fpls.2016.00456)
Supplement: Supplementary file 8 [file Image5.PDF]

Figure S5

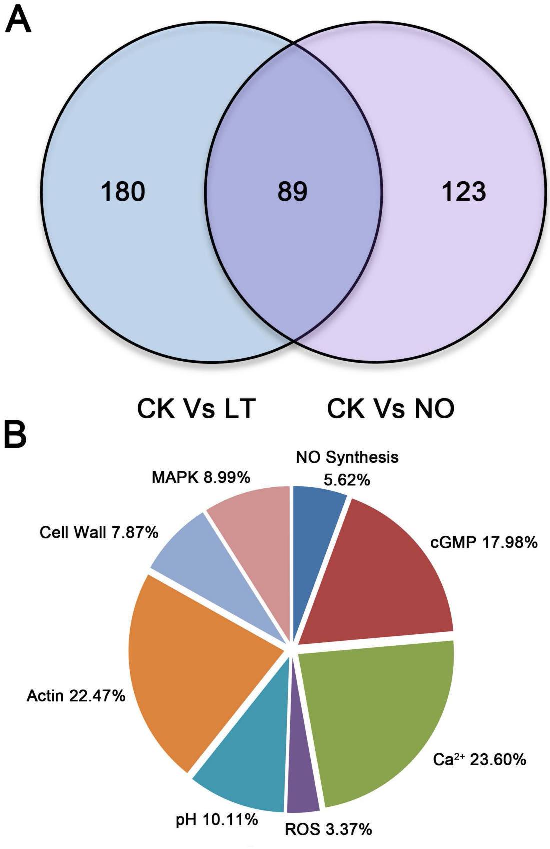

Supplemental Figure 5. The number of signaling pathway-related co-expressed differentially expressed genes (DEGs) identified in CK-VS-LT and CK-VS-NO comparison. A total of 89 DEGs were co-expressed in CK-VS-LT and CK-VS-NO (A), including NO synthesis-related genes, cGMP-related genes, Ca^{2+}-related genes, ROS-related genes, pH-related genes, actin-related genes, cell wall-related genes and MAPK cascade-related genes (B).
