# Supplementary material for: Combined Cytological and Transcriptomic Analysis Reveals a Nitric Oxide Signaling Pathway Involved in Cold-Inhibited Camellia sinensis Pollen Tube Growth
Source: Front Plant Sci. 2016 Apr 14;7:456. doi: 10.3389/fpls.2016.00456 (PMC4830839; doi:10.3389/fpls.2016.00456)
Supplement: Supplementary file 9 [file Image6.PDF]

Figure S6

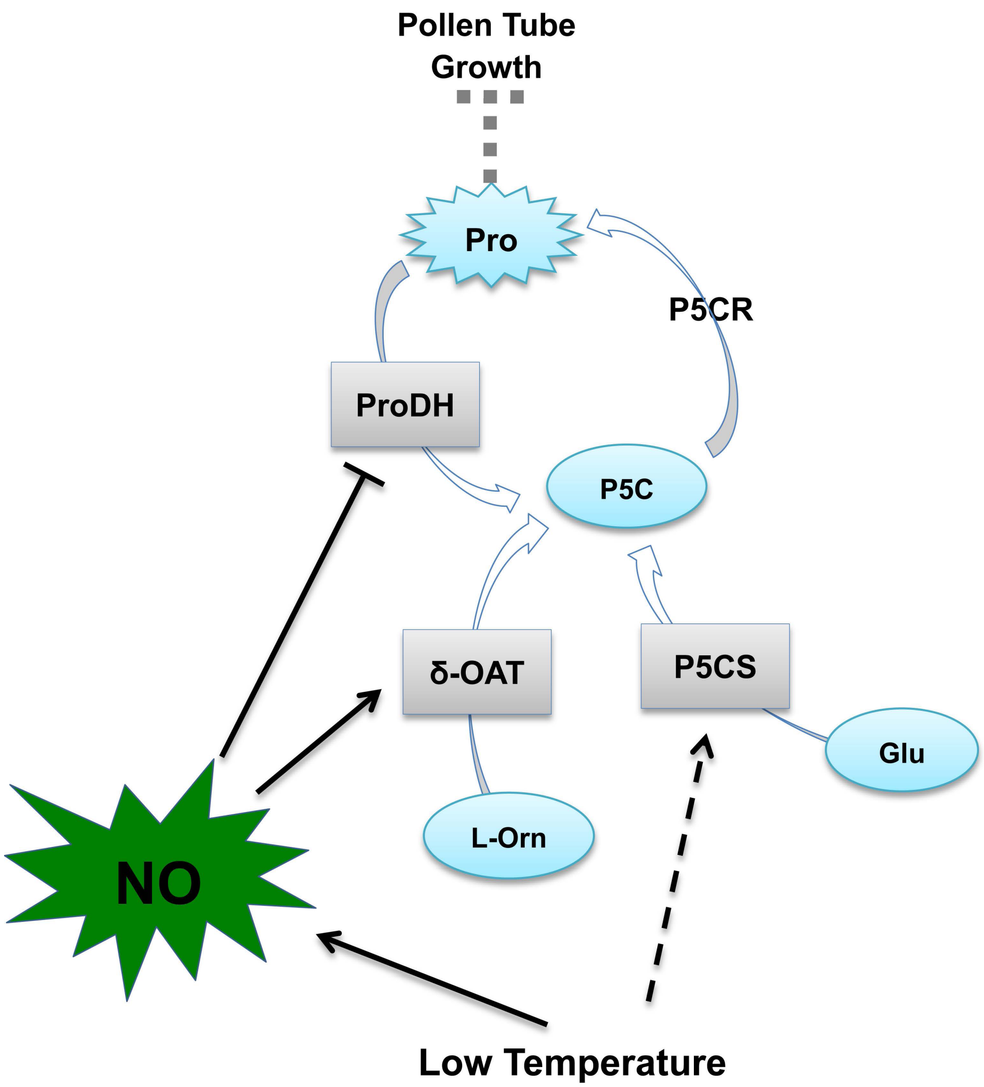

Supplemental Figure 6. Hypothetical model showing the potential mechanism of proline (Pro) accumulation dependent on NO in cold-inhibited *C. sinensis* pollen tube growth. This simplified model was based on the pollen tube models proposed by Wang YH (2012). Cold-induced NO promoted Pro accumulation partly by increasing the expression of *Csδ-OAT* and reducing the expression of *CsProDH* in *C. sinensis* pollen tubes responding to cold stress, which leads to tip growth contraction. P5CS, Δ^1-pyrroline-5-carboxylate synthase; P5CR, pyrroline-5-carboxylate reductase; ProDH, Pro dehydrogenase; δ-OAT, ornithine-δ-aminotransferase.
